# Sugars promote graft union development in the heterograft of cucumber onto pumpkin

**DOI:** 10.1038/s41438-021-00580-5

**Published:** 2021-07-01

**Authors:** Li Miao, Qing Li, Tian-shu Sun, Sen Chai, Changlin Wang, Longqiang Bai, Mintao Sun, Yansu Li, Xing Qin, Zhonghua Zhang, Xianchang Yu

**Affiliations:** 1 Institute of Vegetables and Flowers, Chinese Academy of Agriculture Sciences, Beijing, 100081 China; 2 College of Horticulture, Qingdao Agricultural University, Qingdao, 266109 China; 3 College of Horticulture, Shanxi Agricultural University, Taigu, Jinzhong, Shanxi 030801 China

**Keywords:** Non-model organisms, Cell division

## Abstract

The use of heterografts is widely applied for the production of several important commercial crops, but the molecular mechanism of graft union formation remains poorly understood. Here, cucumber grafted onto pumpkin was used to study graft union development, and genome-wide tempo-spatial gene expression at the graft interface was comprehensively investigated. Histological analysis suggested that resumption of the rootstock growth occurred after both phloem and xylem reconnection, and the scion showed evident callus production compared with the rootstock 3 days after grafting. Consistently, transcriptome data revealed specific responses between the scion and rootstock in the expression of genes related to cambium development, the cell cycle, and sugar metabolism during both vascular reconnection and healing, indicating distinct mechanisms. Additionally, lower levels of sugars and significantly changed sugar enzyme activities at the graft junction were observed during vascular reconnection. Next, we found that the healing process of grafted etiolated seedlings was significantly delayed, and graft success, xylem reconnection, and the growth of grafted plants were enhanced by exogenous glucose. This demonstrates that graft union formation requires the correct sugar content. Furthermore, we also found that graft union formation was delayed with a lower energy charge by the target of rapamycin (TOR) inhibitor AZD-8055, and xylem reconnection and the growth of grafted plants were enhanced under AZD-8055 with exogenous glucose treatment. Taken together, our results reveal that sugars play a positive role in graft union formation by promoting the growth of cucumber/pumpkin and provide useful information for understanding graft union healing and the application of heterografting in the future.

## Introduction

Grafting is a widely used technique to improve horticultural crop product quality, quantity, and disease resistance, with effects on controlling tree vigor and fruit size^1,2^. In production, the achievement of a well-developed graft union determines compatible or incompatible grafting. The reconnections of functional vasculature tissues that differentiate from calli between scions and stocks are essential for compatible grafts^3,4^. In China, approximately 30% of cucumber cultivars are grafted onto pumpkin to overcome soil-borne diseases and increase resistance against abiotic stress, and farmers utilize heterografting methods^5^. There is intricate communication between the two different species at the graft junction^6^. Many research studies have examined the mechanisms of graft union formation based on morphological and metabolic changes^6–8^. However, the molecular development mechanisms of heterografting are less well understood.

Previous work describes a time course for graft formation at the graft junction, including cell adhesion, the formation of a necrotic layer of dead cells, callus formation through cell division, and finally the reconstruction of vascular bundles across the graft junction^4,9^. Calluses, with cells that are close to the cut and attached to the opposing tissue, give rise to cambium, phloem, and xylem^4,10^. Calluses also fill the gaps between adhering tissues to strengthen the adhesion of opposing tissues^11,12^. However, the process of callus formation at the graft interface may be species specific. In grafted *Arabidopsis* hypocotyls, little callus is produced, although in many plants, callus formation is critical for effective grafting^10^. For plant samples that are cut and not grafted, significant amounts of wound-induced calli are formed, and transcriptional analysis indicates that wound healing proceeds via different mechanisms depending on the presence or absence of adjoining tissues^13^. Cell division is rapidly activated during cutting and grafting. Removal of the cotyledons results in the inhibition of cell division and wound healing in cucumber^14^. Sufficient connection of functional vascular bundles between the scion and rootstock is essential for the graft union of woody trees^15,16^. The establishment of phloem connections generally precedes xylem connection at the graft junction in *Arabidopsis*^4^. However, *Arabidopsis* scions that are grafted onto tomato rootstocks can flower and produce seeds in the absence of the scion/stock vascular connection^17^. In addition, plasmodesmata provide an important channel for cell–cell communication at the graft junction, with higher plasmodesmal coupling observed between callus cells than between cortex cells in stem unions of *Prunus* spp.^18^. Despite these important findings, a more detailed study of the anatomy of graft union development is needed for many crops, especially in the field of horticulture.

Plant hormones and sugars accumulate above the cut site and are depleted below due to the severity of vascular tissues^19^. The absolute auxin level is not a key indicator of graft success, but how auxin is perceived may activate auxin responses involving *ABERRANT LATERAL ROOT FORMATION 4* (*ALF4*) and may promote vascular connection at the graft junction^4^. The potential effects of other phytohormones (abscisic acid and salicylic acid) in graft formation are poorly understood, and other molecules (ethylene, jasmonic acids, strigolactones, brassinosteroids, cytokinins, and gibberellins) may have little effect^19^. Sugar accumulation in the grafted top and sugar depletion in the grafted bottom can cause asymmetric expression levels of sugar-induced genes (e.g., ADP-glucose pyrophosphorylase *ApL3*) and sugar-repressed genes (dark inducible 6 *DIN6*, glutamate dehydrogenase 1 *GDH1*, and sugar transporter protein *1 STP1)* in the scion and rootstock. This asymmetric gene expression is a critical feature of grafting and tissue reunion and may play an important role in vascular connections at the graft junction^13^. Adding 0.5% sugar to the grafting medium resulted in faster recovery after grafting and a higher graft success rate compared to 0% sugar plants^20^. Sucrose affects the quantity of calli deposited on sieve plates and further promotes the formation of discontinuous vascular bundles^21^. Sugar transporters *SWEET15* and *SWEET19* activate cell–cell communication between scion and rootstock cells through the CBL interacting protein kinase 14 (*CIPK14*) during the healing process^9^. Recent research demonstrates that β−1,4-glucanase, encoded by the member of the glycosyl hydrolase 9B (GH9B) family (*GH9B3*), is secreted into the extracellular region and functions in cell wall digestion and enables the success of *Nicotiana* interfamily grafting with a diverse range of angiosperms^22^.

Sugars are upstream regulators of the target of rapamycin (TOR), a highly conserved master regulator that plays pivotal roles in controlling cell proliferation, cell size, transcription, photosynthesis, carbon and nitrogen metabolism, and autophagy^23,24^. Only one TOR gene was identified in *Arabidopsis*, and its complex1 (TORC1) consists of TOR kinase, the regulatory-associated protein of TOR (RAPTOR) and lethal with SEC13 protein 8 (LST8). In plants, TOR kinase activity is chemically inhibited by rapamycin, AZD-8055, TORIN 1, and KU-63794^24,25^. The repression of TOR always results in less biomass, smaller leaves and roots, whereas overexpression of TOR or kinase domains also causes abnormalities in *Arabidopsis*, such as severe shoot and inflorescence meristem defects^26^. Therefore, only a moderate increase in the *TOR* expression level (less than 2-fold) can promote root and shoot growth, cell size, and seed yield without visibly affecting plant morphology^27^. In the *Arabidopsis* meristem, glucose and sucrose can activate the S-phase in a CYC-CDK-RBR-independent fashion by activating TOR kinase, which directly phosphorylates E2F (a/b) transcription factors^25^. However, sugar acts independently of TOR activity in promoting maize germination^28^. Additionally, TOR protein kinase is also an energy sensor that regulates cellular homeostasis in balance with sucrose nonfermenting-related kinase 1 (SnRK1), which triggers adaptive responses during carbon/energy deficiency^24^. However, the function of TOR in graft union formation has not yet been studied.

In this study, we performed an anatomical analysis and in-depth RNA-seq analysis to observe the cellular and transcriptional changes that occur during the development of cucumber–pumpkin heterografts. Furthermore, etiolated seedling grafting and the application of exogenous glucose or the TOR inhibitor AZD-8055 demonstrated that sugar and TOR play important roles in cucumber/pumpkin graft union formation. This study provides new insights into the understanding of the mechanism of graft union formation and the application of heterografting in horticultural plants.

## Results

### Reconnection of vascular bundles promotes growth resumption during graft formation

**Fig. 1:**
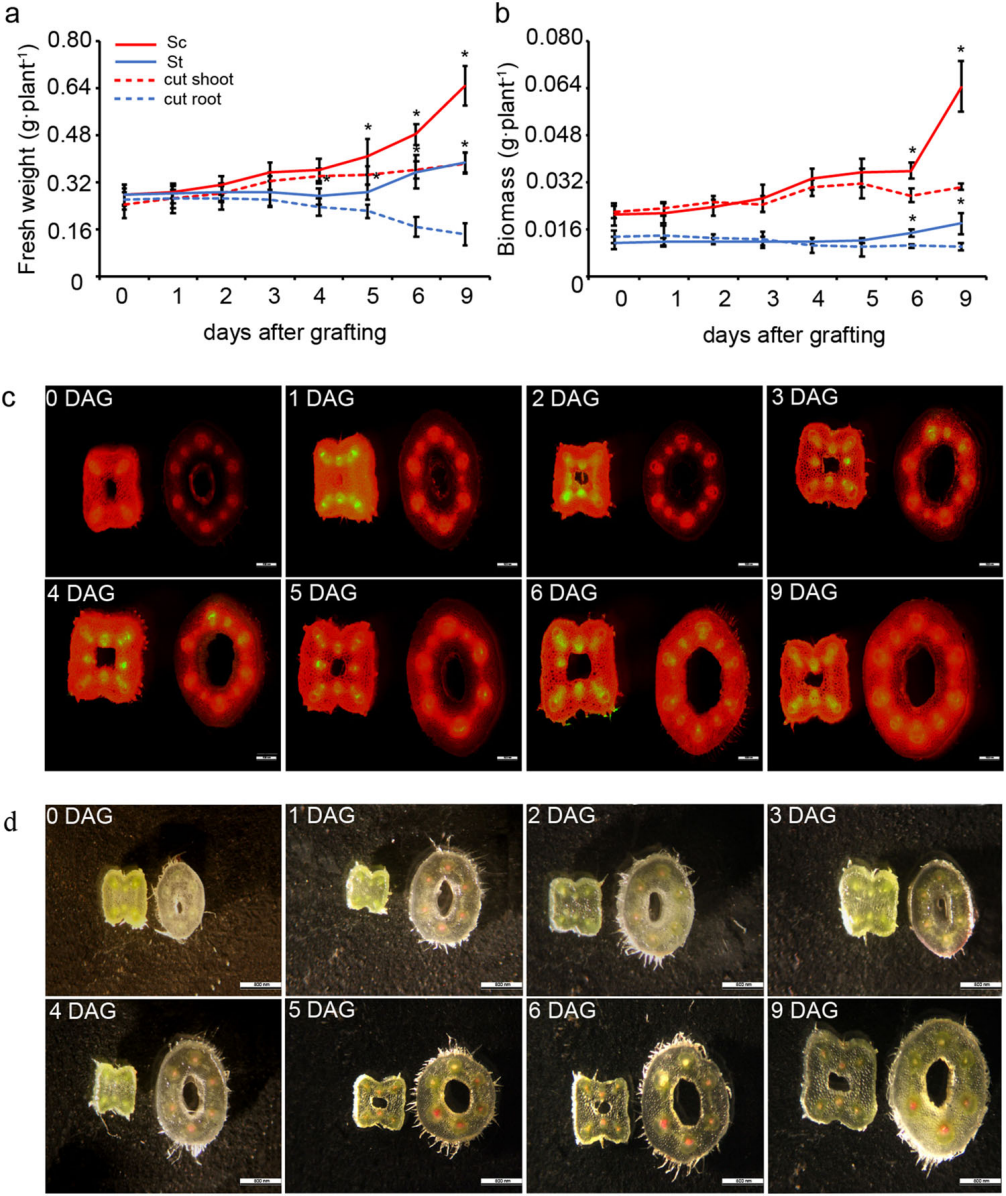
Biomass and reconnection of the phloem and xylem of cucumber–pumpkin plants at 1–9 DAG. **a** Fresh weight. **b** Dry biomass. Sc scion, St rootstock, cut shoot cut shoot (not grafted), cut root cut root (not grafted). Error bars indicate SE (*n* = 8). Asterisks indicate significant differences between Sc and the cut shoot and between St and the cut root, respectively (*t*-test, *P* < 0.05). **c** Phloem connection occurs at 3 DAG. Comparison of the epicotyl of the scion 1 cm above the graft junction with the hypocotyl of the rootstock 1 cm below the graft junction after CFDA application to the cotyledon at 1–9 DAG. **d** Xylem connection occurs at 5 DAG. Comparison of the epicotyl of the scion 1 cm above the graft junction with the hypocotyl of the rootstock 1 cm below the graft junction after the roots were soaked in 0.1% (w/v) acid fuchsin solution at 1–9 DAG. Left is the epicotyl of cucumber, right is the hypocotyl of pumpkin in (**c**, **d**). DAG days after grafting

To obtain heterografted plants, cucumber scions were grafted onto pumpkin rootstocks following previously described protocols^29^ (Fig. S1a). To determine the resumption of growth, the biomass changes during graft union healing were measured by comparing grafted plants (scion and rootstock) and cut plants (both the cut shoot and cut root). Significant biomass increases were found in both the rootstock and scion at 6 days after grafting (DAG), in contrast to the cut shoot and root, respectively (Fig. 1a, b). The carboxy-fluorescein diacetate (CFDA) assay was used to monitor phloem connectivity. We applied CFDA to cotyledons and examined the fluorescent signals on a daily basis (Fig. S1b). By comparison of the epicotyl of the scion 1 cm above the graft junction with the hypocotyl of the rootstock 1 cm below the graft junction, few grafted individuals exhibited a fluorescence signal in the hypocotyl of the rootstock after application of CFDA to the cotyledons at 2 DAG, and by 3 DAG, nearly 85% individuals showed a fluorescence signal (Figs. 1c, S1c). Next, we soaked the rootstock in 0.1% (w/v) acid fuchsin solution and monitored the dye in the epicotyl of the scion 1 cm above the graft junction to assay xylem reconnection (Figs. 1d, S1b). Approximately 90% of scions exhibited acid fuchsin dye at 5 DAG, indicating that the connection of functional vasculature bundles occurred at 5 DAG (Fig. S1c). Taken together, our results showed that phloem reconnection between the scion and rootstock was established two days earlier than xylem recovery, and heterografted plants started growing after xylem reconnection at the graft junction. Additionally, we also compared the graft union healing process between homografts and heterografts (Fig. S2). The results showed that the process of healing is different in cucumber/cucumber, pumpkin/pumpkin, and cucumber/pumpkin. The rapid accumulation of biomass in pumpkin/pumpkin may be due to earlier vascular reconnection compared with cucumber/cucumber and cucumber/pumpkin.

### Anatomical observation and transcriptional analysis during vascular reconnection and healing indicate that the scion and rootstock undergo distinct processes during graft union formation

**Fig. 2:**
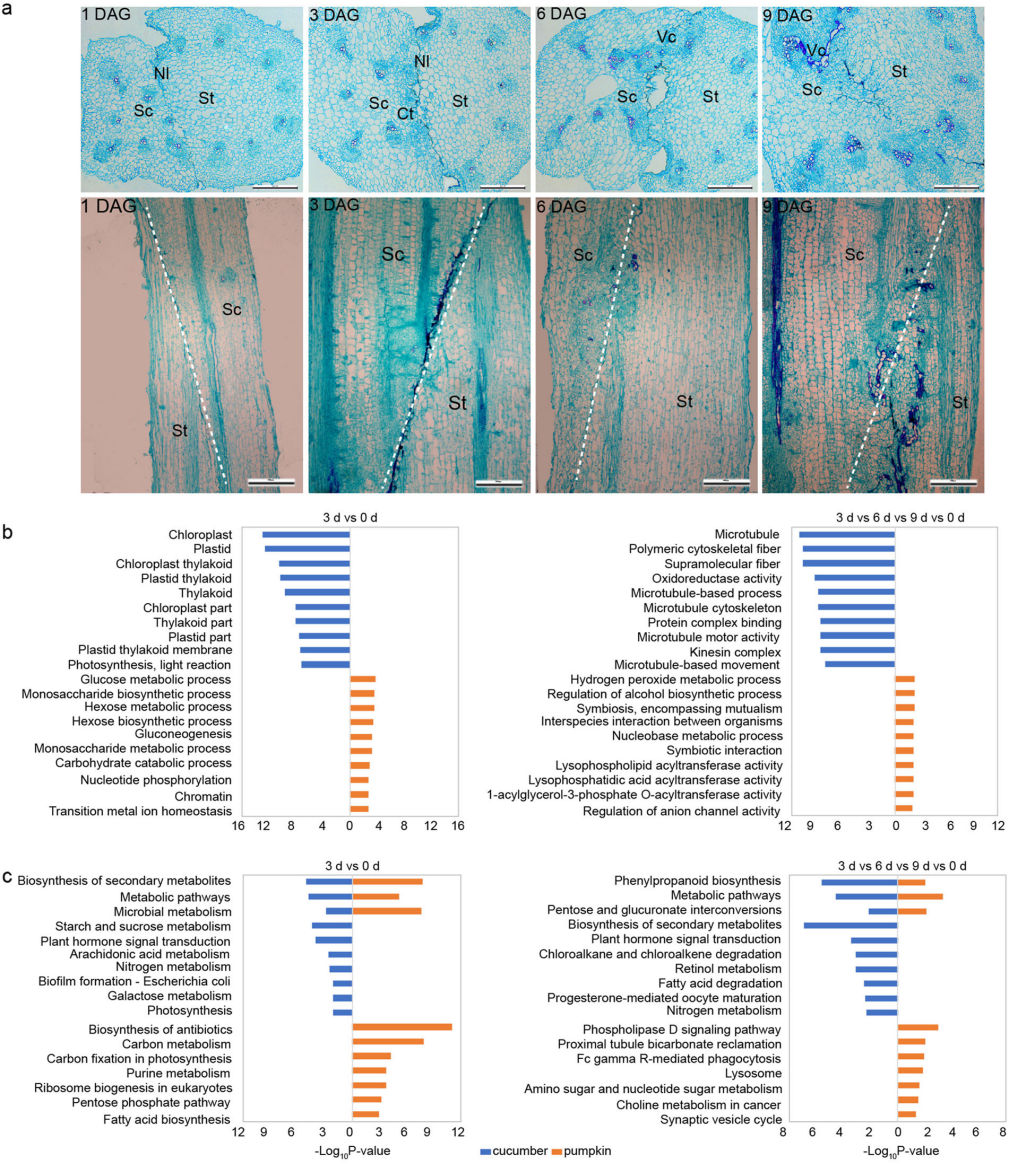
Histological and transcriptome analysis of graft union formation. **a** Transverse and longitudinal sections of the middle part of the graft union were observed at 1, 3, 6, and 9 DAG by the paraffin section method. Sc Scion, Rs Rootstock, NI Necrotic layer, Ct Callus tissue, VB Vascular bundle, Vc Vascular connection, DAG days after grafting. Data represent the means of 5 replicates ± SE. **b**, **c** The 10 most significantly enriched GO categories (**b**) and KEGG pathways (**c**) of DEGS only significantly expressed in comparison of 3 d vs 0 d and commonly shared in comparisons of 3 d vs 0 d, 6 d vs 0 d, 9 d vs 0 d in cucumber and pumpkin

To study graft union formation, the combinations between cucumber and pumpkin in the hypocotyls were transversely cut, and anatomical analyses were performed at 1, 3, 6, and 9 DAG (Fig. 2a). Transverse sections were cut in the middle part of the graft union, where more vascular reconnection occurred (Fig. S3a). The necrotic layer was observed at the graft junction at 1 DAG. At 3 DAG, the callus tissues appeared on the scion side, the rootstock had no evident morphological changes, and the necrotic layer still partly existed. Vascular tissue connections between the scion and stock were observed at 6 DAG, and the scion and rootstock exhibited different developmental patterns. At 9 DAG, more connections were observed, and more reconnected vascular development was found on the scion side of the graft junction (Fig. 2a). Additionally, we also found that the diameter of the reconnected vasculature was larger than that of other adjacent vessels (Fig. S3b, c).

The transcriptional profile differences in the scion and rootstock during graft union formation were compared using RNA-seq (Fig. S4). Differentially expressed genes (DEGs) were identified as genes with absolute fold change >2 and false discovery rate (FDR) < 0.01. The qRT-PCR results of 12 randomly selected DEGs indicate that our RNA-Seq data and the criteria for DEGs are reliable (Fig. S5). In total, 4269 and 8634 DEGs were identified during graft formation in the scion and rootstock, respectively (Fig. S4c, Table S1). At 3 DAG, approximately 21.5% of the pumpkin genes were differentially expressed, but only 11.9% of the cucumber genes showed differential expression. However, both the scion and rootstock had fewer differentially expressed genes (approximately 7%) at 6 or 9 DAG than at 0 DAG (Fig. S4b).

To further investigate the specific responses in the scion and rootstock during graft union formation, we performed a gene ontology (GO) enrichment analysis of the DEGs for the comparisons of 3 d vs 0 d, 6 d vs 0 d, and 9 d vs 0 d in the scion and rootstock. Genes that were only differentially expressed at 3 DAG or 6 DAG in the cucumber scion were enriched in the “chloroplast”, “plastid”, “thylakoid”, “cell wall organization or biogenesis”, and “cell wall macromolecule metabolic process” pathways (Figs. 2b, S6). In the pumpkin rootstock, genes that were differentially expressed only at 3 DAG or 6 DAG were enriched in different pathways, including “phosphoenolpyruvate carboxykinase (ATP) activity”, “glucose metabolic process”, “exopeptidase activity”, “motor activity”, and “carbohydrate catabolic process” (Figs. 2b, S7). Additionally, genes that were only differentially expressed at 9 DAG in the scion were enriched in cell structure-related GO terms, such as “golgi apparatus” and “regulation of cell cycle phase transition” (Figs. S6, S7). GO enrichment analysis of the overlapping DEGs that were differentially expressed at any time point after grafting was also performed. Consistently, in cucumber scions, these DEGs were enriched in pathways that are important to cell structure formation or maintenance, such as “microtubule”, “polymeric cytoskeletal fiber”, and “supramolecular fiber”, while in the rootstock, the DEGs were enriched in metabolism-related GO terms, such as “hydrogen peroxide metabolic process”, “regulation of alcohol biosynthetic process”, and “symbiosis, encompassing mutualism” (Fig. 2b). Interestingly, these results support our anatomical observation, in which the scion exhibited more vigorous development.

The KEGG analysis also showed a significantly distinct response between the scion and rootstock (Figs. 2c, S8, S9). At 3 DAG, “biosynthesis of secondary metabolites”, “metabolic pathways”, and “microbial metabolism” were enriched in both the scion and rootstock (Fig. 2c). Additionally, sugar metabolism-related KEGG pathways were downregulated in both the scion and rootstock at 3 DAG, including “starch and sucrose metabolism”, “carbon metabolism”, “fructose and mannose metabolism”, and “carbon fixation in photosynthetic organisms”. KEGG analysis of the overlapping DEGs showed that three pathways were enriched in both the scion and rootstock, i.e., “phenylpropanoid biosynthesis”, “metabolic pathways”, and “pentose and glucuronate interconversions” (Fig. 2c). It is worth noting that “plant hormone signal transduction” pathways were only enriched in the scion, i.e., not in the rootstock, before vascular bundle reconnection (Figs. 2c, S8 and S9). Taken together, the results revealed distinct activities between the scion and rootstock during both vascular reconnection and healing, and sugar metabolism may play an important role during graft union formation.

### The change in the sugar content during graft union formation

**Fig. 3:**
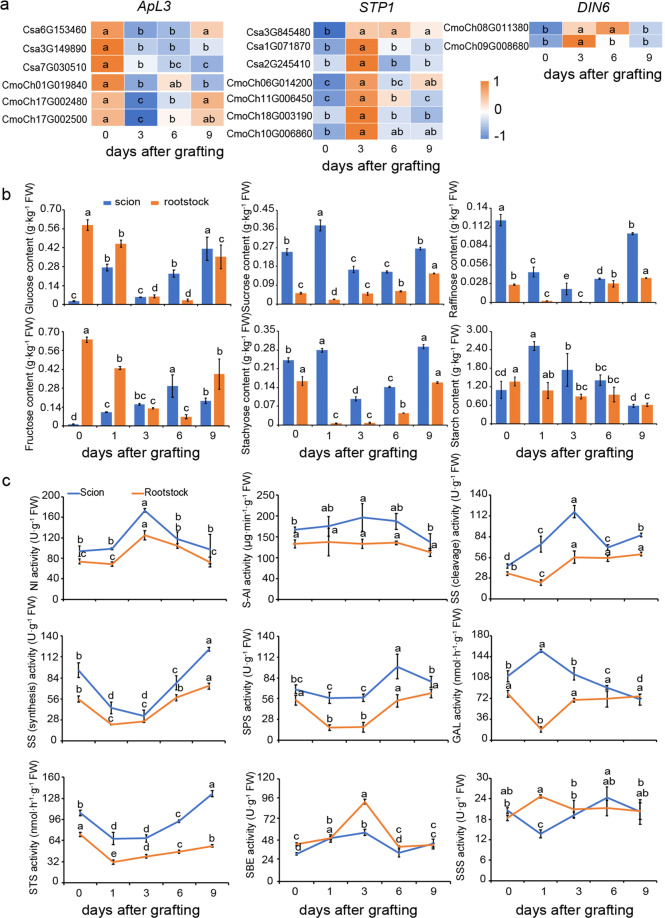
Expression pattern of sugar responsive genes, sugar content, and sugar metabolic enzyme activity genes during the graft union formation. **a** Heatmap of gene expression for DEGs (*ApL3, STPI, DIN6*) related to sugar response. Heatmap color indicates the fold change of expression. **b** Content of glucose, fructose, sucrose, stachyose, raffinose, starch in the hypocotyl of the scion and rootstock at the graft junction at 0, 1, 3, 6, 9 days after grafting. **c** The enzyme activity of sucrose metabolic enzymes (S-AI, NI, SPS, and SS in the synthesis and degrading directions), RFO metabolic enzymes (GAL, STS) and starch metabolic enzymes (SBE, SSS) in the hypocotyl of the scion and rootstock at the graft junction at 0, 1, 3, 6, and 9 days after grafting. S-AI soluble acid invertase, NI neutral invertase, SPS sucrose phosphate synthase, SS sucrose synthase, GAL α-galactosidase, STS stachyose synthase, SSE starch branching enzyme, SSS soluble starch synthase, RFO raffinose family oligosaccharides. Error bars indicate SE (*n* = 3). Different letters indicate significant differences (one-way ANOVA, *P* < 0.05)

To study sugar metabolism changes during graft union formation, we first analyzed the expression levels of the sugar-induced gene *ApL3* and the sugar-repressed genes *STP1*, *DIN6*. We found decreased expression of *CsApL3* and increased expression of *CsSTP1* in both the scion and rootstock at 3 DAG. In addition, the expression of *CsDIN6* was significantly increased only in the rootstock, i.e., not in the scion (Fig. 3a). Thus, the sugar responses were similar in the scion and rootstock of the heterograft, and sugar starvation was maintained at the junction during the healing process. This is consistent with changes in sugar levels during the healing process in which the levels of sucrose, stachyose, and raffinose were decreased significantly in the hypocotyl of the scion and rootstock at 3 DAG or 6 DAG compared with 0 DAG (Fig. 3b). However, glucose, fructose, sucrose, stachyose, and starch accumulated in the hypocotyl of the scion at 1 DAG, and the levels of these sugars (all but starch) decreased markedly in the rootstock at 1 DAG (Fig. 3b). Moreover, the levels of glucose and fructose were increased in the scion but decreased in the rootstock at 3 DAG and 6 DAG. Additionally, the starch levels did not exhibit any significant changes at 3 DAG and 6 DAG and then declined at 9 DAG (Fig. 3b).

The activities of enzymes involved in sugar metabolism were also examined during graft union formation (Fig. 3c). The activities of sucrose synthase (SS) in the synthesis direction and sucrose phosphate synthase (SPS) decreased dramatically at 1 DAG at the graft junction, whereas SS activity in the degradation direction, soluble acid invertase (S-AI) and neutral invertase (NI) showed no distinct changes, indicating that the observed sucrose accumulation in the scion may be caused by physical obstruction of the junction. The activities of sucrose synthesizing enzymes (SS in the synthesizing direction and SPS) decreased at 3 DAG and then exhibited a significant increase at 6 DAG. In contrast, the activities of sucrose-degrading enzymes (all but S-AI) showed the opposite trend (Fig. 3c), consistent with the levels of sucrose at the graft junction.

In addition, the α-galactosidase activity increased significantly in the scion and decreased in the rootstock at 1 DAG, followed by a rapid recovery at the graft union. Stachyose synthase (STS) activity showed similar development patterns in the scion and rootstock, with marked decreases from 1 DAG to 6 DAG, which potentially caused decreased raffinose and stachyose levels during graft union development (Fig. 3b). The activity of soluble starch synthase (SSS), which was involved in starch biosynthesis, was decreased in the scion and increased in the rootstock at 1 DAG and were maintained at a steady level during graft union healing. However, the activity of starch branching enzyme (SBE) involved in starch degradation was increased during graft union development. This might be responsible for the lower levels of starch at 9 DAG (Fig. 3b). These results clearly showed that sugars accumulated in the scion at 1 DAG, suggesting a significant need for energy for healing at 3 DAG.

### The levels of sugars affect graft union formation

**Fig. 4:**
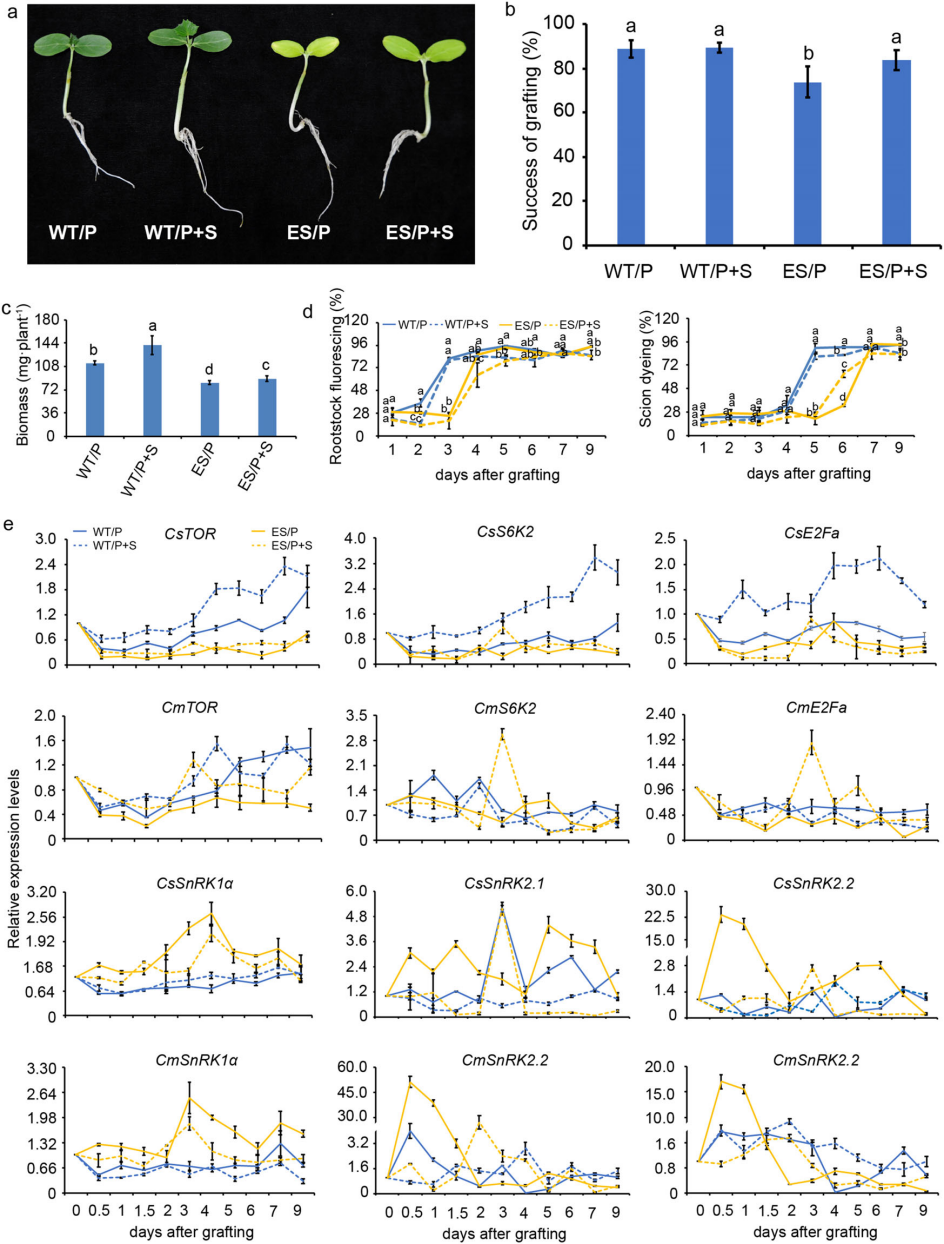
Effect of sugar levels on graft union formation. **a** Phenotype of the cucumber/pumpkin and etiolated cucumber/pumpkin with/without application of exogenous glucose at 4 DAG. 25–30 plant replicates for every treatment. **b** The graft efficiency was determined 13 days after grafting. **c** The biomass was determined 13 days after grafting. **d** Phloem connection and xylem connection, which was assessed by CFDA and acid fuchsin as described in Fig. 1. **e** Expression pattern of genes related to the TOR pathway, including *TOR, S6K2, E2Fa, SnRK1α, SnRK2.1, SnRK2.2*. WT/P the normal cucumber grafted onto pumpkin, WT/P+S the normal cucumber onto pumpkin under 0.5% glucose treatment, ES/P the etiolated cucumber grafted onto pumpkin, ES/P+S the etiolated cucumber grafted onto pumpkin under 0.5% glucose treatment, DAG days after grafting. Error bars indicate SE (*n* = 3). Different letters indicate significant differences (one-way ANOVA, *P* < 0.05)

To investigate the role of sugar levels in this process, we sprayed a range of concentrations of exogenous glucose solution (0, 0.2, 0.5, 1, and 5%) on the scion after grafting. The results showed that phloem reconnection was not significantly different under exogenous glucose treatments, and xylem reconnection occurred at 4.5 days after grafting with 0.5% exogenous glucose. However, the graft efficiency was lowered by higher levels of exogenous glucose (Fig. S10a, b). Next, we used etiolated cucumber seedlings, which have lower sugar levels than normal seedlings (green seedlings) (Fig. S10c, d), as scion material and conducted an exogenous glucose assay with or without 0.5% glucose solution. The results showed that the grafted etiolated seedlings grew slowly, the new cambium in the scion was not formed at 3 DAG (Figs. 4a, c, S11), and both phloem and xylem connections were delayed (occurring at 4 DAG and 7 DAG, respectively) (Fig. 4d). However, these biological processes were improved by the presence of exogenous glucose, including graft efficiency and biomass, and reduced the degree of etiolation (Fig. 4a–c). The phloem reconnection of the scion and rootstock was not affected by exogenous sugar in either the normal seedlings or the etiolated one. Compared with grafted etiolated tissue without sugar, the xylem connection was achieved 1 day earlier by the addition of 0.5% glucose, whereas there was no significant change in the normal tissue with exogenous sugars compared with the normal tissue without sugar treatment. Additionally, the growth of the scions was significantly enhanced by the application of exogenous glucose during graft union healing, especially the normal tissue in the presence of glucose (Fig. 4b, c). Our results suggested that the appropriate level of sugar is not only necessary for graft union formation but also critical for grafted plant growth during healing.

To further study the role of sugars at the graft junction, we next analyzed the expression levels of sugar signaling genes, including the regulator of growth factors *TOR*, small ribosome protein S6 kinase (*S6K2)*, and E2F transcription factors (*E2Fa*), and the energy response factors *SnRK1α* and *SnRK2*. As shown in Fig. 4e, compared with that at 0 DAG, the expression of *CsTOR*, *CsS6K2*, and *CsE2Fa* was significantly downregulated in the scion of normal heterografts (WT/P) during graft union formation, whereas the expression levels of these genes were significantly increased with the application of exogenous glucose (Table S2). In contrast, in the scion of etiolated heterografts, *CsTOR*, *CsS6K2*, *CsE2Fa* were significantly downregulated during the healing process, and exogenous glucose only partially affected the expression patterns (Fig. 4e, Table S2). In the rootstock, exogenous sugar slightly affected the expression of *CmTOR*, *CmS6K2*, *CmE2Fa* in the normal heterografts but played a significant role in changing the expression pattern of these genes in the etiolated heterografts (Fig. 4e). The energy response factor *SnRK1α* exhibited a similar expression pattern in the scion and rootstock; it was significantly downregulated at the graft junction of the normal heterografts but upregulated in the etiolated heterografts (Table S2). However, exogenous sugar significantly reduced the upregulated expression level of *SnRK1α* during graft union formation in etiolated heterografts (Fig. 4e). Additionally, the levels of sugar signal genes *CsSnRK2.1* and *CsSnRK2.2* were increased earlier in the etiolated heterografts than in the normal heterografts, but they were triggered rapidly in both heterografts at the early period of graft union formation, which was significantly activated within 4 DAG (Fig. 4e). Exogenous sugars can downregulate the expression of *SnRK2.1* and *SnRK2.2*. For instance, in etiolated heterografts, the expression of *SnRK2.2* was delayed and reduced by the application of exogenous glucose (Fig. 4e). These results implied that sugars play a positive role in graft union development and the growth of heterografts, possibly via TOR pathways.

### Effects of the TOR inhibitor AZD-8055 on graft union formation

**Fig. 5:**
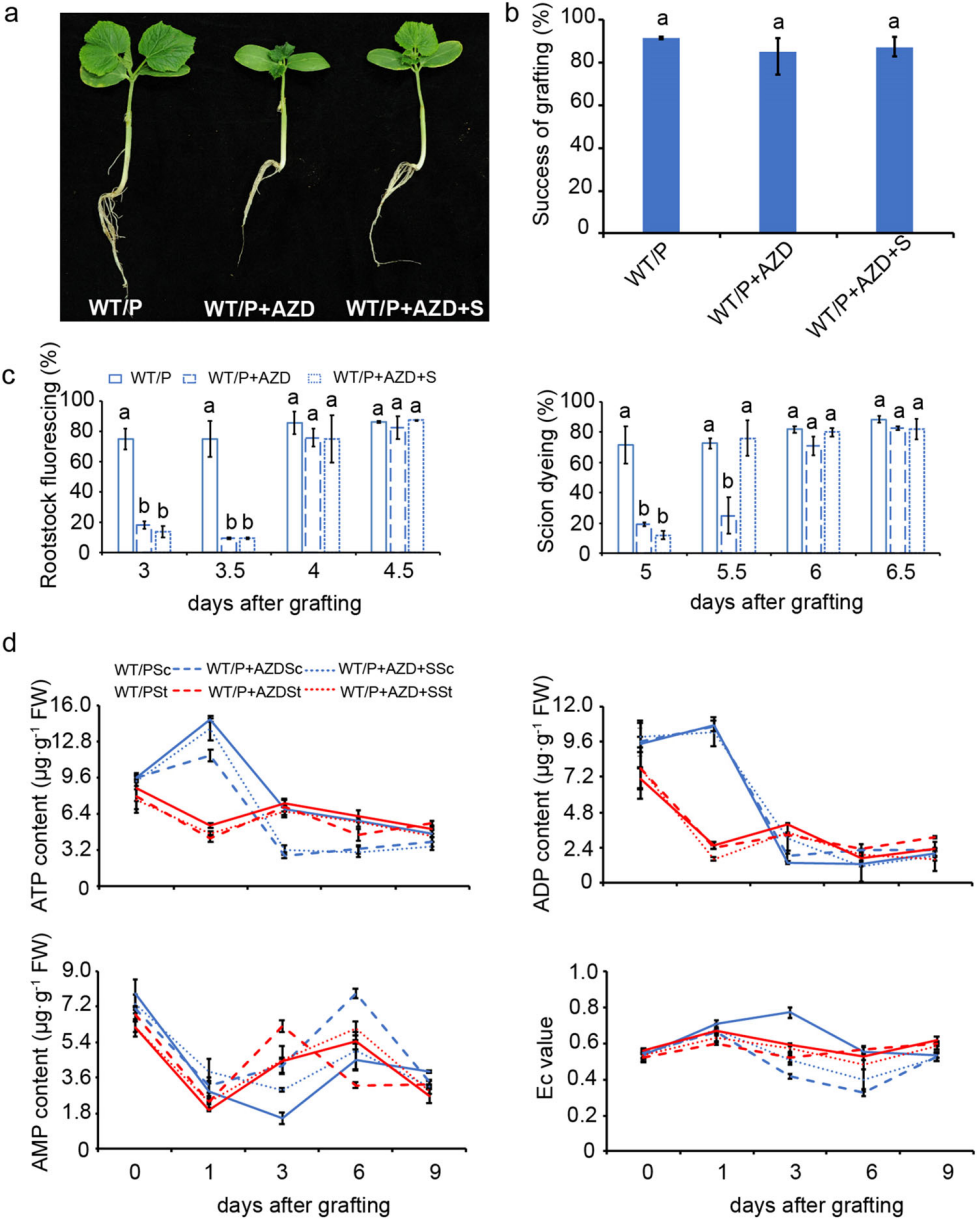
Effect of the TOR inhibitor AZD-8055 and exogenous glucose on the graft union formation. **a** Phenotypes of cucumber/pumpkin with/without inhibitors or in the presence or absence of exogenous glucose at 13 days after grafting. 30–45 plant replicates for every treatment. **b** The graft efficiency was counted at 13 days after grafting. **c** Phloem connection and xylem connection, which was assessed at 5, 5.5, 6, and 6.5 days after grafting by CFDA and acid fuchsin as described in Fig. 1. **d** The levels of ATP, ADP, AMP, and energy charge in the hypocotyl of scion and rootstock during graft union formation. WT/P the normal cucumber grafted onto pumpkin, WT/P+AZD the normal cucumber grafted onto pumpkin under AZD-8055 treatment, WT/P+AZD+S the normal cucumber grafted onto pumpkin under AZD-8055 and 0.5% glucose treatment. WT/PSc the scion of the normal cucumber grafted onto pumpkin, WT/PSt the rootstock of the normal cucumber grafted onto pumpkin, WT/P+AZDSc the scion of the normal cucumber grafted onto pumpkin under AZD8055 treatment, WT/P+AZDSt the rootstock of the normal cucumber grafted onto pumpkin under AZD8055 treatment, WT/P+AZD+SSc the scion of the normal cucumber grafted onto pumpkin under AZD8055 and 0.5% glucose treatment, WT/P+AZD+SSt the rootstock of the normal cucumber grafted onto pumpkin under AZD8055 and 0.5% glucose treatment. Error bars indicate SE (*n* = 3). Different letters indicate significant differences (one-way ANOVA, *P* < 0.05)

The TOR kinase, as a master regulator, integrates nutrients and energy signals to promote cell proliferation and growth^25^. To study the role of TOR kinase in graft union formation, we chose the second-generation specific TOR inhibitor AZD-8055, which significantly inhibited cucumber growth at a concentration of 10 μM, in our experiment (Fig. S12). Graft success was not significantly affected by exogenous AZD-8055 in the presence or absence of sugars, but the growth of grafted seedlings was inhibited by AZD-8055, and exogenous sugars partially complemented this inhibitory effect (Fig. 5a, b). Consistently, the vascular connection was also delayed by 0.5 days by AZD-8055, and only xylem reconnection was partially recovered by exogenous sugars (Fig. 5c). To determine the status of energy metabolism during graft union formation, the levels of ATP, ADP, and AMP at the graft junction were measured. The results showed that ATP and ADP levels were significantly higher at 1 DAG and subsequently decreased in the scion, whereas both ATP and ADP maintained lower levels in the rootstock after grafting. After AZD-8055 application, the content of ATP in the scion was significantly decreased, whereas it remained similar to untreated seedlings in the rootstock. The lower levels of ATP can be partially recovered by exogenous glucose in the scion. The ADP level was not significantly affected in the scion and rootstock by AZD-8055 with or without glucose (Fig. 5d). The AMP levels remained lower at the graft junction of the normal group compared with those in the inhibitor-treated group and were also improved by treatment with AZD8055 and glucose. Therefore, the lower energy charge was maintained at the graft junction after application of AZD-8055 compared with that in the normal group, and this change was slightly reduced by exogenous sugars (Fig. 5d, Table S3).

**Fig. 6:**
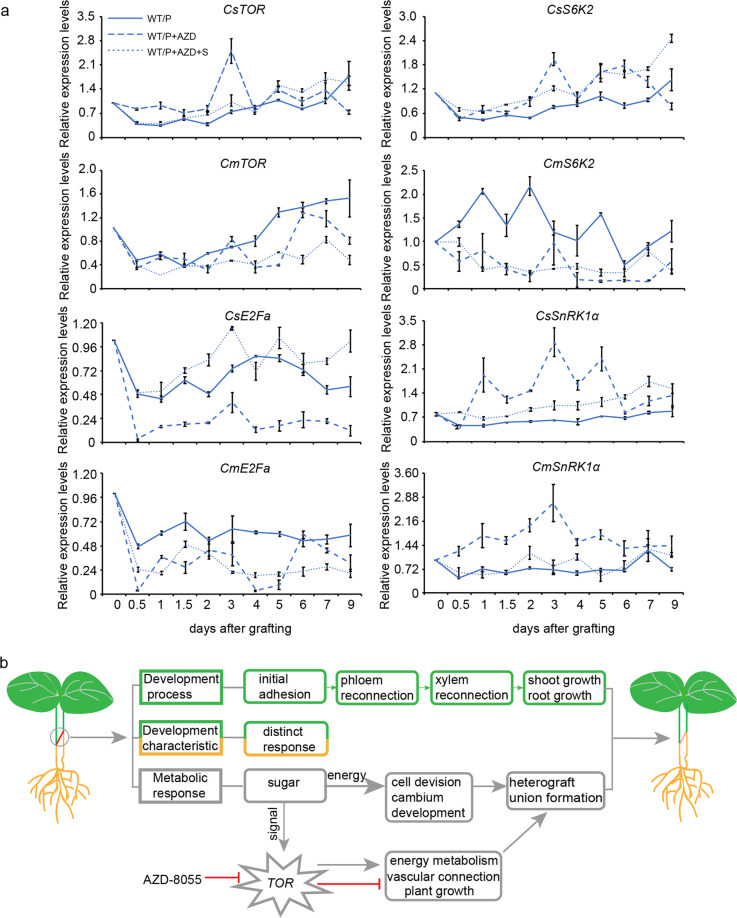
A model summarizing the graft union development in the heterograft of cucumber onto pumpkin. **a** Expression patterns of genes related to the TOR pathway. **b** a putative model for graft union formation of heterografts. WT/P the normal cucumber grafted onto the normal pumpkin, WT/P+AZD the normal cucumber grafted onto the normal pumpkin treated by AZD-8055, WT/P+AZD+S the normal cucumber grafted onto the normal pumpkin treated by both AZD-8055 and exogenous glucose

To investigate whether TOR signaling pathways are involved in graft union formation, we also checked the expression of *TOR*, *S6K*, *E2Fa*, and *SnRK1α* in this process (Fig. 6a, Table S4). Interestingly, the expression of *CsTOR* and *CsS6K2* was higher under TOR inhibitors with/without exogenous glucose treatments, whereas *CmTOR* and *CmS6K2* were markedly downregulated under these treatments. The expression of *E2Fa* was significantly inhibited by AZD-8055 in both the scion and rootstock; in contrast, *SnRK1α* was triggered at the graft junction, and the upregulation of *SnRK1α* was slightly relieved by exogenous sugars. Taken together, these results indicated that the glucose-TOR (Glc-TOR) signaling pathways possibly regulated graft union formation by balancing growth conditions and energy metabolism.

## Discussion

Graft union formation is an interesting and complex process^9^. Previous studies have shown that this process is species specific, and there are significantly different responses between homografts and heterografts in *Arabidopsis* and grapes^6,13,30^. In this study, we performed an anatomical study and in-depth RNA-seq analysis using cucumber–pumpkin heterografts to observe the cellular and transcriptional changes that occur during the development of heterografts. We conclude the results in Fig. 6b and propose that the Glc-TOR signaling pathway may play a positive role in graft union formation and cucumber/pumpkin growth.

### Reconnection of vascular bundles prior to rootstock growth resumption during graft union formation

Cucumber and pumpkin have well-developed vascular bundles, making them ideal experimental materials to measure the grafting process and better understand graft union formation. Previous work described the stages of graft union development, including the production of a necrotic layer, cell proliferation into calli, and connection of vascular bundles^7,17,29,31^. In this study, the functional phloem connection occurred two days earlier than the xylem connection (Fig. 1c, d). However, the biomass of the rootstock did not immediately increase after phloem reconnection, with significant growth starting only after xylem connection (Fig. 1a, b). This is different from what is reported for grafted *Arabidopsis*^9,26^. Additionally, we also measured the biomass and reconnection of homografted plants (Fig. S2). More biomass accumulated in pumpkin/pumpkin than in grafted cucumber/cucumber and cucumber/pumpkin during graft union formation. The reconnection of phloem and xylem was at 4 DAG and 7 DAG, respectively, in homografted cucumber, and this was achieved at 3 DAG and 4 DAG, respectively, in homografted pumpkin (Fig. S2). This demonstrated that the process of graft union formation was obviously different between the homografted and heterografted plants. Together, our results indicated that the scion and rootstock simultaneously affect the growth of heterografted plants during graft union formation; however, the development process of the scion and rootstock varies for different plant species.

### Graft union formation exhibited different responses between the scion and rootstock of heterografts

Wounding and grafting resulted in significant callus formation. In our study, evident callus was produced in the scion but not in the rootstock during heterograft union formation (Fig. 2a), which might play a role in filling the gaps and seal the wound^32^ or may subsequently differentiate to form epidermal, mesophyll, and vascular tissues^26^. These sequential events have been described for woody perennial species, but in some other species, there was little callus formation in the graft zone during graft union formation^6,9^. Moreover, it is difficult to distinguish callus and original cambium dedifferentiation into secondary growth.

Asymmetric expression of specific genes is essential to tissue reunion. For example, in cut *Arabidopsis* inflorescence stems, *PAP2.6* *L* is exclusively expressed in the upper region, and *ANAC071* is exclusively expressed in the lower region^33^. In a recent study, transcriptome dynamics analysis showed that at the graft junction of homografted *Arabidopsis*, many genes associated with cambium, phloem, and xylem development and the sugar response were more highly expressed on one side than on the other, and this asymmetry disappeared after reconnection of the vascular tissues^13^. Our transcriptome analysis also revealed a significantly distinct response between the scion and rootstock, as evidenced by DEGs, enriched GO and KEGG pathways, and changes in the levels of key genes involved in cambium formation and cell division. A total of 12% of the cucumber genes and 22% of the pumpkin genes showed differential expression at 3 DAG. The data suggested more dynamic metabolism in the rootstock than in the scion, suggesting that the rootstock is not a passive partner for healing. The asymmetric number of DEGs between the scion and rootstock disappeared at 6 DAG and 9 DAG (Fig. S4b). This result was not consistent with observed changes for grafting in *Torreya grandis*, with more DEGs in the rootstock than in the scion for most metabolic pathways at both 7 DAG and 14 DAG^34^. This inconsistency may be caused by the longer healing process in woody plants compared to herbaceous plants. Nevertheless, functional vascular reconnection may help the scion and rootstock of heterografts become integrated. However, GO- and KEGG-enriched terms of DEGs exhibited a notable difference between the scion and rootstock during vascular reconnection. Scions and rootstocks may recognize adjoining tissues and activate different wound healing mechanisms than cut and separated tissues during homograft union formation^13^. At the same time, heterografting may upregulate stress responses at the graft junction compared with the responses for nonself rootstocks^6^. Additionally, vascular development, cell proliferation and division, and hormone-related genes also triggered distinct responses between the scion and rootstock (Figs. S13a, S14, Table S5). Therefore, the scion and rootstock may trigger different healing metabolic activities during graft union formation after detecting each other, and the precise process mostly depends on the species.

### Sugar metabolism is active and plays an important role during graft union formation

Sugars, as respiratory substrates, are essential for the generation of energy, and as metabolic intermediates, they are required for the synthesis of macromolecules^35^. Sugar response correlates with asymmetric gene expression in the scion and rootstock during graft union formation in *Arabidopsis*. A total of 20–31% of asymmetrically expressed genes can respond to sugars and exhibit changes in expression patterns from asymmetry to symmetry after phloem reconnection^13^. Based on our data, there was significantly enriched expression of genes involved in sugar metabolism in the scion and rootstock during graft union formation (Fig. S13b). Additionally, there was marked accumulation of sugars (glucose, fructose, sucrose, stachyose, and starch) in the scion at 1 DAG, with lower sugar levels at 3 and 6 DAG, and the enzymes of sugar metabolism were also changed (Fig. 3a). These results indicate that graft union healing is an energy-consuming process. In addition, sugars also act as activators of important cell division and elongation processes at the homograft junction^13^. If carbon availability is sufficient for the completion of a developmental program, sugar signals can regulate developmental transitions^36^. In our study, the expression of sugar-sensing genes (*CsApL3*, *CsSTP1*, and *CsDIN6*) and sugar levels exhibited a sugar-starvation response at the graft junction at 3 DAG. Sugar starvation affects several central aspects of development by regulating photosynthetic activities and carbon remobilization (Fig. 2b, c). A higher or lower exogenous sucrose level can reduce the graft survival rate of *Arabidopsis*^13,20^. In our study, the higher concentration of exogenous glucose reduced graft efficiency, whereas the lower concentration had no significant effect on the graft survival rate but improved the growth of grafted plants during the healing process. Moreover, we found that etiolated cucumber/pumpkin exhibited a lower survival rate and delayed speed of graft union formation (Fig. 4b, d). However, this effect could be partially complemented by the spraying of exogenous glucose, including the survival rate, xylem reconnection, and de-etiolation. Additionally, the growth of grafted plants was significantly increased by exogenous glucose, especially that of normal plants. These results demonstrated that sugars are not only indispensable for graft union formation but also play a positive role in promoting the growth of grafted plants.

### The Glc-TOR pathway may be involved in heterograft union formation

Sugar signaling can be regulated by protein kinases related to energy metabolism^23^. TOR kinases are master growth regulators that modulate nutrient status and energy signaling to promote cell proliferation and growth in plants^37^. TOR also works as a critical integrator of light and sugar signals to drive postembryonic growth and response to stress^38^. The term glucose-TOR (Glc-TOR) signaling was first used to explain the normal TOR activity by glucose through glycolysis and mitochondrial bioenergetics to reprogram the transcriptome and activate meristems^25^. Depletion of endogenous sugars led to inhibition of TOR activity, whereas exogenously applied glucose reactivated TOR activity^25^. In this study, *TOR* expression levels were downregulated compared with those at 0 DAG and were upregulated after phloem connection during graft union formation. Exogenous glucose had a positive effect, whereas lower endogenous sugar had a negative effect on *TOR* expression (Fig. 4e). Consistently, the two major conserved downstream targets of TOR signaling, S6K2, which phosphorylates RPS6 and might finally promote protein translation, and E2Fa, which promotes the expression of S-phase and cell cycle progessions^23^, had a similar response to sugars during graft union formation (Fig. 4e). The TOR kinase complex also interacts closely with and in opposite ways to SnRK1 kinase in the regulation of nutrient-driven processes, and SnRK1α is responsible for the majority of SnRK1 kinase activity, which is activated to induce a stress response under sugar and energy starvation^23^. In our study, *SnRK1α* was significantly upregulated in etiolated cucumber seedlings/pumpkin and was downregulated by exogenous sugars. Additionally, the expression of *SnRK2* had a trend similar to that of *SnRK1α*, and *SnRK2s* work in close connection with *SnRK1* in nutrient stress mitigation^39^. These results demonstrate that the TOR signaling pathway was triggered and regulated by exogenous glucose during the healing process. However, its role in the vascular bundle connection or grafted plant growth is not known.

AZD-8055 is a specific TOR kinase activity inhibitor and is always used to discover TOR functions in plants^40^. In our study, the spraying of 10 μM AZD-8055 inhibited cucumber growth, whereas the inhibitory effect was not obvious in rapamycin-sprayed treatments (Fig. S12). Graft efficiency was not affected by the spraying of AZD-8055, whereas vascular bundle reconnection was delayed and the growth of grafted plants was inhibited by AZD-8055. Moreover, the energy charge was lower at the graft junction after inhibitor treatment during the healing process, and this could be partially complemented by the spraying of exogenous glucose. Decreased TOR activity and lower energy charge are possibly the main reasons for the slow growth phenotype^41^. Sugar (glucose and sucrose) is the upstream signal for TOR activation, and the effect of TOR inhibitors could not be recovered by the feeding of exogenous sugars^42^. In our study, exogenous sugar still promoted xylem reconnection and growth of grafted plants under AZD-8055 treatment. We speculated that sugars might promote plant growth independently of the Glc-TOR pathways. Consistent with research on maize germination, Glc stimulation of growth requires TOR kinase during the first 48 h, and Glc could also have a positive effect on germination with the TOR inhibitor AZD-8055^28^. Strikingly, *TOR* and *S6K* expression was significantly upregulated by AZD-8055 in the scion, especially at 3 DAG. On the one hand, the degree of *TOR* transcript repression does not always fully correlate with the amount of active TOR protein, which is affected by posttranscriptional regulation^43,44^. In addition, only the appropriate increases in the expression of *TOR* levels (less than 2-fold) positively stimulate root and shoot growth, and strong overexpression of full-length *TOR* results in developmental abnormalities^27^. Therefore, the precise regulation of both temporal and spatial *TOR* expression is critical and complicated for the growth of plants. However, *E2Fa* and *SnRK1α* were significantly downregulated and upregulated, respectively, by the application of AZD8055. These results indicated that the Glc-TOR pathway might play a role in promoting vascular reconnection and the growth of grafted plants during graft union formation. How TOR regulates graft union formation requires further study.

In conclusion, we present a model (Fig. 6b) with graft union developmental changes of heterografts. Root growth resumption occurs after the connection of both the phloem and xylem during graft union formation. Changes in anatomy and genome-wide gene expression in the scion and rootstock exhibited significant distinct responses. A low sugar supply is maintained in the scion and rootstock during graft union development, and the Glc-TOR pathway may play an important role in the vascular connection and growth of grafted plants. The findings of our work provide useful information for understanding cucumber/pumpkin graft union formation.

## Materials and methods

### Plant materials and grafting

Cucumber (*Cucumis sativus* L.) and pumpkin (*Cucurbita moschata* Dutch.) were planted in an artificial chamber at the Institute of Vegetables and Flowers Chinese Academy of Agricultural Sciences, Beijing, China. Plants were cultivated with a day/night (12/12 h) cycle at 25 °C/18 °C and 60–70% relative humidity. For etiolated cucumber, cucumber seeds were germinated on moisture filter paper in the dark at 28 °C for 2 days, and germinated seedlings were transferred to a growth chamber filled with vermiculite and grown in light incubators maintained at 28 °C:15 °C (day:night) under dark conditions with 50–60% humidity. The etiolated cucumber was readied for grafting when cotyledons had fully opened (6 d after sowing). The cucumber was grafted onto the pumpkin by splice grafting (Fig. S1a). The grafted seedlings were then maintained under the conditions described previously^29^. Graft unions were harvested at 0, 3, 6, and 9 DAG (Fig. S4a), and the 0 DAG sample was harvested immediately after grafting (less than 2 min). Three biological replicates were performed for each time point. Samples were harvested and immediately transferred to liquid nitrogen before storage at −80 °C before further use.

### Biomass determination and assays of phloem and xylem connectivity

Cut and separated shoot, cut and separated root, and grafted plant samples that were separated into scion and rootstock 1-10 DAG were cleaned with deionized water and weighed. The samples were placed in an oven at 105 °C for 15 min and then dried at 75 °C to constant weight. Eight to fifteen plants were weighed for each experiment, and three replicates were performed for each time point. Phloem and xylem connectivity were measured by CFDA and acid fuchsin movement across the graft union, respectively (Fig. S1b). To assay the phloem connection, 1 mg CFDA was dissolved in 10 μL DMSO, and then 4.6 μL of this solution was added to 50 ml MS solution (pH 6.5–6.7) to obtain a final 1 mM CFDA solution. The cotyledon center of the scion was gently scraped with a sharp single-edge razor to create a small opening for CFDA to enter. Next, a 2 μL CFDA sample was added per cotyledon, and fluorescence in rootstock hypocotyls was measured after 1 h incubation^26^. For xylem connection, plant roots were incubated in a solution of 0.1% (w/v) acid fuchsin, and the cucumber hypocotyls were examined after 40 min^17^.

### Paraffin section microscopy

The samples were collected at 0, 1, 3, 6, and 9 DAG. The collected samples were placed in FAA for 2 days and then dehydrated through an ethanol/xylene series followed by a xylene/paraplast series. The samples were then embedded in paraplast and sectioned as described previously^45^. Samples were sectioned to 10 μm vertically using a rotary microtome (KE3390; KEDEE; Zhejiang, China), dewaxed, rehydrated, cleaned, stained with Fast Green, counterstained with safranin, and then fixed with neutral balata. Sections were examined using a light microscope (BX53; Olympus Corp., Tokyo, Japan), and representative sections were photographed.

### RNA extraction, library construction, and sequencing

Total RNA was isolated from different samples using RNeasy plant mini kits (TianGen, China) according to the manufacturer’s instructions and treated with RNase-free DNase I (Takara, Japan) to degrade genomic DNA. The quality and quantity of RNA were checked by a Nanodrop 1000 Spectrophotometer (Thermo Fisher Scientific, Wilmington, DE) and Agilent 2100 Bioanalyzer (Agilent Technologies, Santa Clara, CA). A total of 3 μg RNA per sample was used for cDNA library preparation. Sequencing libraries were constructed by the NEBNext^®^ UltraTM RNA Library Prep Kit for Illumina^®^ (NEB, Ipswich, MA, USA) following the manufacturer’s instructions. Briefly, the steps included mRNA enrichment, mRNA fragmentation, second-strand cDNA synthesis, size selection, and PCR amplification as previously described^46^. The prepared libraries were then sequenced on an Illumina HiSeq 2000 platform (San Diego, CA, USA).

### cDNA sequence assembly and functional annotation

To ensure the accuracy of the data, low-quality reads were filtered by FastQC software. Qualified reads were mapped to the reference sequences using HISAT2 with the default parameters^47,48^. The reference genome and gene database were downloaded from an open genomic resource platform (http://cucurbitgenomics.org)^49^. The reads were then assembled through StingTie^48^. Based on protein database annotations (NCBI nonredundant (NR), Swiss-Prot, Kyoto Encyclopedia of Genes and Genomes), sequences with the highest similarities were classified by KEGG and Gene Ontology (GO) using Blast2GO. The sequenced raw data in our study were deposited in the NGDC GSA database (https://bigd.big.ac.cn/gsub/submit/gsa, accession code: CRA003782).

### Analysis of differentially expressed genes (DEGs)

The gene expression level was calculated based on TPM (transcripts per million). The DEGs of the scion/rootstock combination at 3 d vs 0 d, 6 d vs 0 d, and 9 d vs 0 d after grafting were identified as genes with absolute fold change >2 and FDR < 0.01. Homology gene function prediction was performed using the *Arabidopsis* database (http://www.arabidopsis.org/index.jsp). DEGs significantly enriched in functional classifications or pathways at 3 d vs 0 d, 6 d vs 0 d, and 9 d vs 0 d were identified through GO and KEGG enrichment analyses.

### Determinations of carbohydrate and sugar metabolic enzyme activities

Soluble carbohydrates were extracted and assayed according to previous reports^50,51^ with minor modifications. Samples (2 g FW) were harvested from the scion part 3 cm above the graft junction and rootstock part 3 cm below the graft junction at 0, 1, 3, 6, and 9 d after grafting. Samples were extracted three times in 80% (v/v) ethanol for 45 min at 80 °C. The extracts were combined, decolorized with activated carbon, and evaporated to dryness at 40 °C with applied vacuum. The dried extracts were redissolved in 500 μL distilled water and filtered through a 0.45 μm filter. Next, 20 μL samples were analyzed for the carbohydrate content using high-performance liquid chromatography (HPLC) (Agilent 2100 system) and a Sugar-Pak I column (Waters, 6.5 mm × 300 mm, 10 mm). Separation was performed at 75 °C with water as the eluent at 0.5 mL min^−1^ and a refractive index detector (G1362A RID Agilent USA). Eluted sugars were identified and quantified based on retention time and peak heights of sugar standards (Sigma). Our study specifically focused on the contents of sucrose, raffinose, galactinol, and stachyose. Starch content was measured using a Starch Content Assay Kit (Solarbio, Cat# BC0700) following the manufacturer’s protocols.

STS activity was determined according to a previous report^50^ with minor modifications. Samples (2 g FW) were finely ground in a chilled mortar with four volumes of chilled extraction buffer containing 50 mM HEPES–NaOH (pH 7.0) and 20 mM 2-mercaptoethanol. The extract was then centrifuged at 28,000 × *g* for 20 min. The resulting supernatant was used for the STS activity assay after being dialyzed with 20 mM HEPES-NaOH buffer (pH 7.0) containing 20 mM 2-mercaptoethanol for 16 h at 4 °C. Dialyzed extract (100 μL) was added to 100 μL reaction buffer containing 50 mM HEPES-NaOH (pH 7.0), 20 mM 2-mercaptoethanol, 10 mM galactinol, and 40 mM raffinose. Normals were similar but lacked galactinol. Reaction mixtures were typically incubated at 25 °C for 90 min and then terminated by the addition of 100 μL of 0.1 M NaOH. The mixtures were heated for 30 s in a boiling water bath and cooled to 25 °C for 40 min in the presence of 2 mM NAD, 0.1 U myo-inositol dehydrogenase, and 50 mM Na_2_CO_3_ (pH 9.5) in a total reaction volume of 1.0 mL. The reduction of NAD was measured spectrophotometrically at 340 nm.

Other enzyme activities were assayed by corresponding assay kits from Solarbio Life Sciences (Beijing, China) according to the manufacturer’s protocols. Briefly, enzyme activities involved in sugar metabolism (sucrose, stachyose, starch) were assayed by a sucrose synthase (decomposition direction SS-I) activity detection kit (Solarbio, Cat#BC4310), S-AI detection kit (Solarbio, Cat#BC2470), NI activity detection kit (Solarbio, Cat#BC0570), SS activity detection kit (Solarbio, Cat#BC0580), sucrose phosphate synthase (SPS) activity detection kit (Solarbio, Cat#BC0600), α-galactosidase (GAL) activity detection kit (Solarbio, Cat#BC2570), SSS activity detection kit (Solarbio, Cat#BC1850), and SBE activity detection kit (Solarbio, Cat#BC1860), with spectrometric analyses.

### Exogenous glucose and TOR-specific inhibitor treatments

To test the effect of sugars on grafting, the grafted cucumber/pumpkin was sprayed with different concentrations of glucose (0, 0.2, 0.5, 1, 5%) (W/V) from 0 to 7 days after grafting. Fifty plant replicates were performed for every treatment. To test the effect of TOR inhibitors (rapamycin and AZD-8055) on cucumber, we chose two treatment modes, spraying or adding them to the nutrient solution. When the cotyledon of cucumber had just opened, one part was sprayed with a range of concentrations of rapamycin (0, 5, 10 μM) and AZD-8055 (0, 1, 10 μM) for 5 days. The other parts were transferred to a 5 L (33 cm 33 5 cm 33 5 cm) plastic tank filled with ½-strength Hoagland’s nutrient solution, and a range of concentrations of rapamycin (0, 5, 10 μM) were added after 3 days. The phenotype was observed after 5 days. Fifteen plant replicates were performed for every treatment. For the grafted cucumber, we sprayed with 10 μM AZD-8055 with/without 0.5% glucose 0–7 days after grafting, and normal cucumber was sprayed with water. Rapamycin and AZD-8055 were stored and dissolved in DMSO according to the manufacturer’s instructions.

### Determinations of ATP, ADP, and AMP contents and energy charge

Samples (0.2 g FW) were harvested from the scion part 3 cm above the graft junction and rootstock part 3 cm below the graft junction at 0, 1, 3, 6, and 9 days after grafting. ATP, ADP, and AMP levels were extracted and assayed according to previous reports^52^. ATP, ADP, and AMP measurements were performed on a Waters 2695 high-performance liquid chromatograph (Waters, Inc., Milford, MA, USA) using a Diamonsil ll-C18 column (250 × 4.6 mm, 5 μm) and an ultraviolet detector at 254 nm. Sample aliquots of 10 µl were injected into the HPLC. The concentrations were calculated according to the external standard program. The energy charge (EC) was calculated as [ATP + 1/2ADP]/[(ATP + ADP + AMP)].

### Quantitative real-time PCR analysis

RNA preparations were extracted as described above in triplicate with three biological replications using the RNAprep Pure Plant Plus Kit (Tiangen, Beijing, China). First-strand cDNA synthesis was performed using a FastQuant cDNA Synthesis kit (Tiangen, Beijing, China) according to the manufacturer’s instructions. All gene-specific primers were designed using Primer 5.0 (PREMIER Biosoft International, Canada) (Table S6), and qRT-PCR was performed using a DyNAmo Flash SYBR Green qPCR kit (Thermo, USA) and a CFX96 qPCR System (Bio-Rad, USA). Expression levels were calculated using the 2^−ΔΔCt^ method and were normalized to the level of the reference gene *LEA26*^53^.

### Statistical analysis

All the data are the average values of at least three replicates and their standard deviations. The statistical evaluations were performed via one-way analysis of variance, followed by individual comparisons with Duncan’s multiple range tests (*P* < 0.05) using SPSS software.

## Supplementary information

Sugars promote graft union development in the heterograft cucumber onto pumpkin

Table S1 . The list of DEGs in RNA-seq analysis during graft union formation
