# Supplementary material for: Sugars promote graft union development in the heterograft of cucumber onto pumpkin
Source: Hortic Res. 2021 Jul 1;8:146. doi: 10.1038/s41438-021-00580-5 (PMC8245404; doi:10.1038/s41438-021-00580-5)
Supplement: Supplementary file 1 — Sugars promote graft union development in the heterograft cucumber onto pumpkin [file 41438_2021_580_MOESM1_ESM.pdf]

Supplementary Information

Sugars promote graft union development in the heterograft cucumber onto pumpkin

Authors: Li Miao, Qing Li, Tian-shu Sun, Sen Chai, Changlin Wang, Longqiang Bai, Mintao Sun, Yansu Li, Xin Qin, Zhonghua Zhang*, Xianchang YU*

Fig. S1 The grafting methods (a), sketch map for phloem and xylem reconnection assaied by CFDA and acid fuchsin (b), and phloem and xylem reconnection at the graft union (c).

Fig. S2 Biomass (a) and reconnection of vasculature bundles (b) at the graft union in homograft plants and heterograft plants.

Fig. S3 Histological analysis of graft union formation.

Fig. S4 Sample location and transcriptome analysis for graft union healing.

Fig. S5 Validation of RNA-seq expression patterns by quantitative reverse transcription-PCR for 12 selected DEGs.

Fig. S6 Enriched GO categories for DEGs which were only significantly expressed in comparisons of 3 d vs 0 d, 6 d vs 0 d and 9 d vs 0 d in cucumber.

Fig. S7 Enriched GO categories for DEGs which were only significantly expressed in comparisons of 3 d vs 0 d, 6 d vs 0 d and 9 d vs 0 d in pumpkin.

Fig. S8 Enriched KEGG pathways of DEGs which were only significantly expressed in comparisons of 3 d vs 0 d, 6 d vs 0 d and 9 d vs 0 d in cucumber.

Fig. S9 Enriched KEGG pathways of DEGs which were only significantly expressed in comparisons of 3 d vs 0 d, 6 d vs 0 d and 9 d vs 0 d in pumpkin.

Fig. S10. Effect of glucose on the graft union formation and the levels of sugars in normal cucumber and etiolated cucumber.

Fig. S11 Transverse sections of graft union at 3 DAG and 6 DAG in WT/P and ES/P, respectively.

Fig. S12 Effect of rapamycin and AZD-8055 on the cucumber growth.

Fig. S13 Overview of expression patterns of DEGs in genes related to cambium development and cell division (a) and sugar metabolism (b) at the graft junction.

Fig. S14. Expression patterns of hormonal signaling-related DEGs at graft union formation.

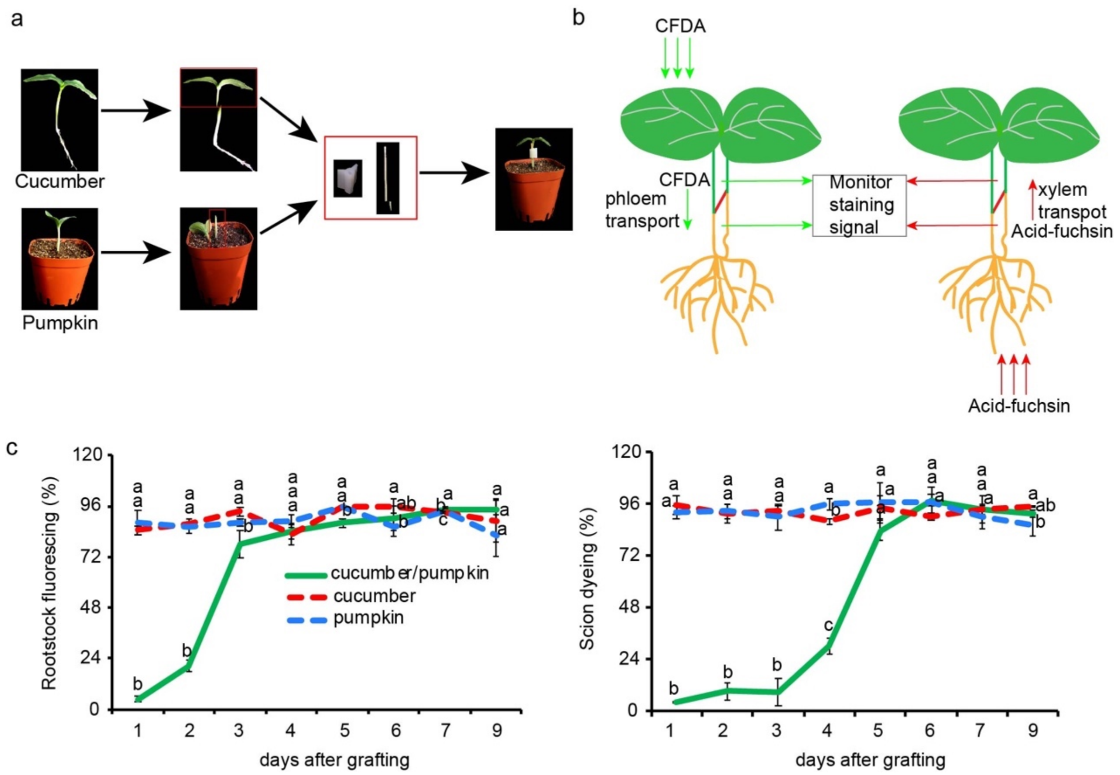

Fig. S1 The grafting methods (a), sketch map for phloem and xylem reconnection assaied by CFDA and acid fuchsin (b), and phloem and xylem reconnection at the graft union (c). a Scions were sown 2 d earlier than rootstocks. When cotyledons of the scion had fully opened (6 d after sowing) and the cotyledon of the rootstock had started to open (4 d after sowing), the cotyledon and growing point of rootstocks were removed and a 30° angled cut was made on the hypocotyl of the rootstocks and scion with a razor blade. The scion hypocotyl was then spliced to the hypocotyl of the rootstock using a plastic tube. **b** For assaying phloem reconnection, CFDA was applied to scion and fluorescence was monitored in the hypocotyl of scion and rootstock, respectively. Similarly, for assaying xylem reconnection, acid fuchsin was applied to root and signal was monitored in the hypocotyl of scion and rootstock, respectively. **c** Phloem reconnection occurs at 3 DAGs, as most of individuals was monitored the fluorescence in the rootstock, and xylem reconnection occurs at 5 DAGs, as most of individuals was observed the acid-fuchsin dyeing in the scion. Different letters indicate significant differences (one-way ANOVA, $P < 0.05$).

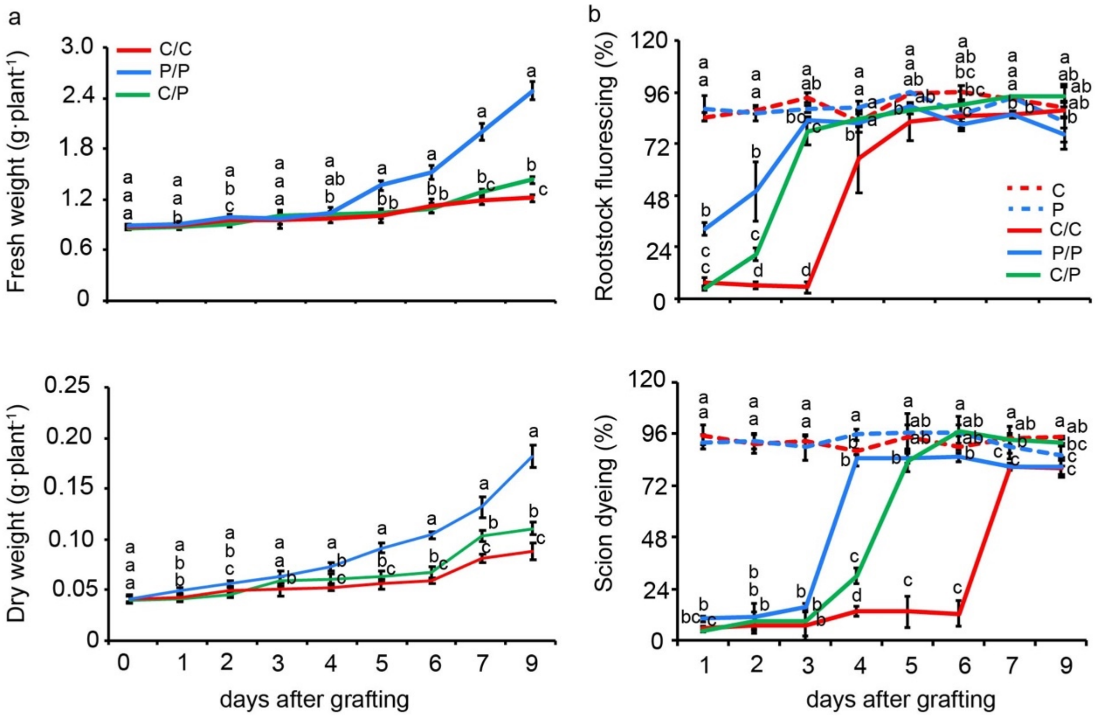

Fig. S2 Biomass (a) and reconnection of vasculature bundles (b) at the graft union in homograft plants and heterograft plants. Phloem reconnection and xylem reconnection were assayed as described in **Fig. S1b**. C, the normal cucumber; P, the normal pumpkin; C/C, the normal cucumber grafted onto the normal cucumber; P/P, the normal pumpkin grafted onto the normal pumpkin; C/P, the normal cucumber grafted onto the normal pumpkin. Different letters indicate significant differences (one-way ANOVA, $P < 0.05$). 8-21 plant replicates for every treatment.

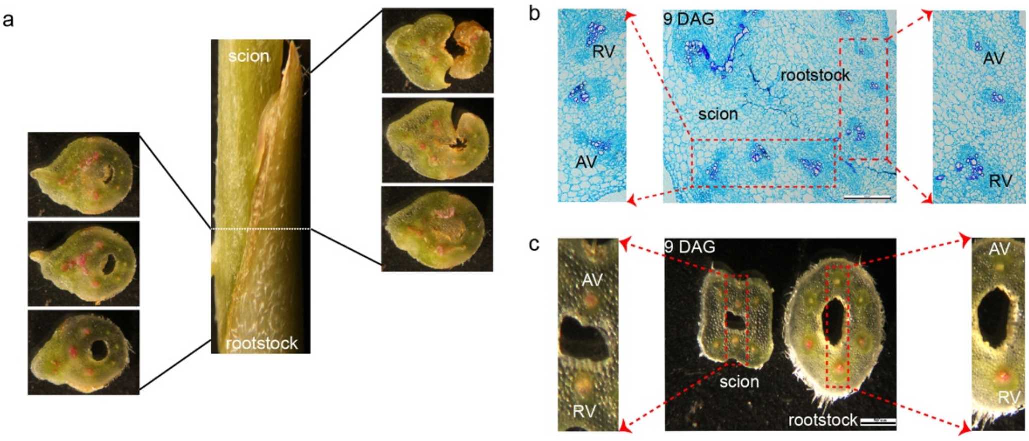

Fig. S3 Histological analysis of graft union formation. a Transverse morphology of graft union at 9 DAG of hand-sliced section after the roots soaked in acid fuchsin solution. **b** Transverse sections of the middle part of the graft union at 9 DAG. **c** Xylem reconnection at 9 DAG. Compare the epicotyl of scion 1cm above the graft junction with the hypocotyl of rootstock 1cm below the graft junction after the roots were soaked in acid fuchsin

solution. RV, reconnected vascular tissue; AV, adjacent vascular tissue. DAG, days after grafting. Data represent the means of 5 replicates ± SE.

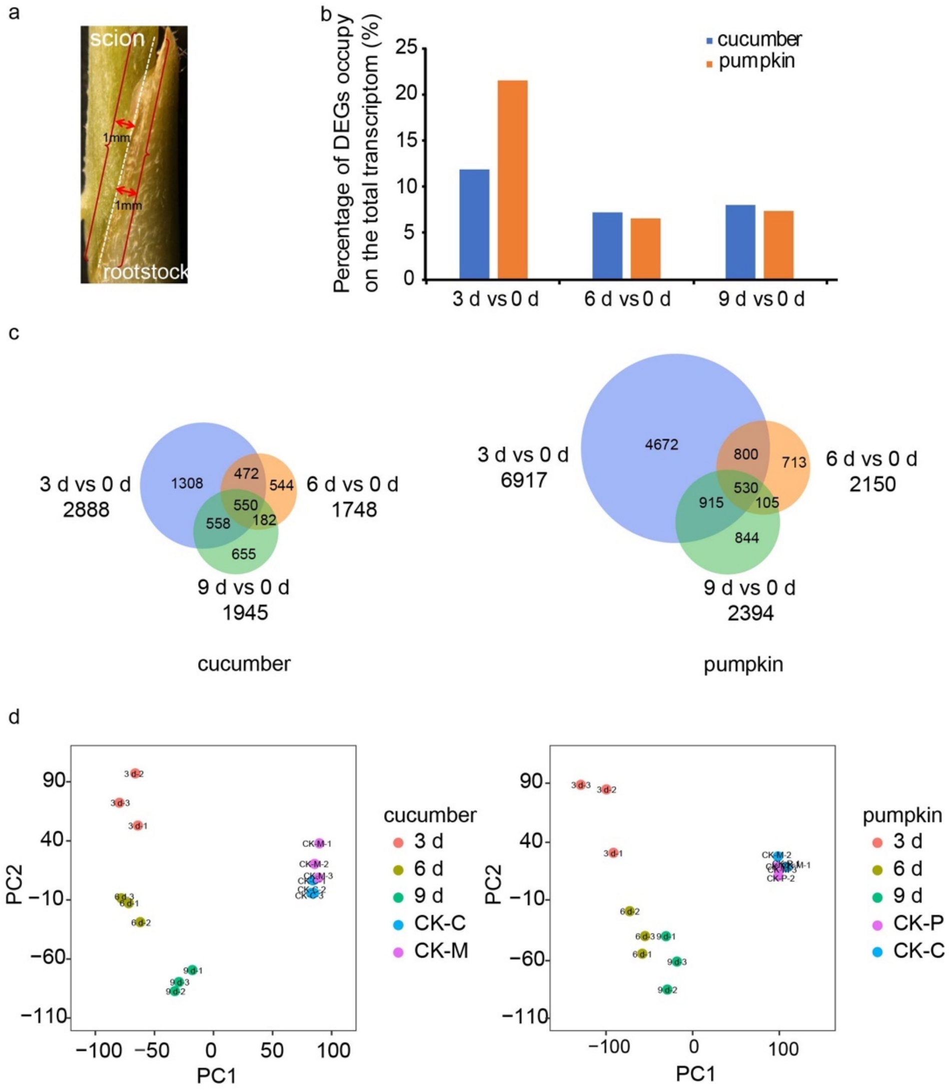

Fig. S4 Sample location and transcriptome analysis for graft union healing. a Graft union tissues were trimmed to between 1 mm above and 1 mm below the cut site and harvested at 0 , 3 , 6 , and 9 days after grafting. **b** The percentages of DEGs in the total transcriptome for comparisons of 3 d vs 0 d, 6 d vs 0 d, and 9 d vs 0 d in scion and rootstock, respectively. **c** Venn diagrams of DEGs in comparisons of 3 d vs 0 d, 6 d vs 0 d in cucumber and pumpkin, respectively. **d** PCA of expression data shows clustering of similar samples. PCA, principal component analysis. “CK-C” and “CK-P” represent cucumber and pumpkin samples were harvested rapidly after grafting, respectively. “CK-M” represents samples were harvested by mixing pumpkin and cucumber after grafting.

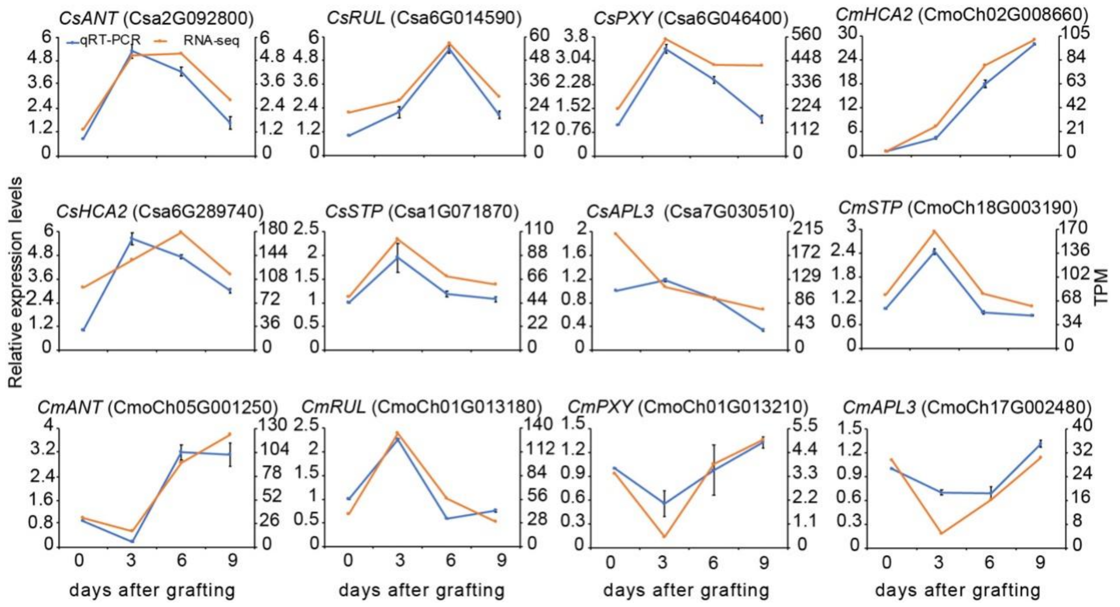

Fig. S5 Validation of RNA-seq expression patterns by quantitative reverse transcription-PCR for 12 selected DEGs. The left Y-axis indicates the expression level (TPM) of RNA-seq, and the right Y-axis indicates the expression level by qRT-PCR. Data represent 3 independent replicates.

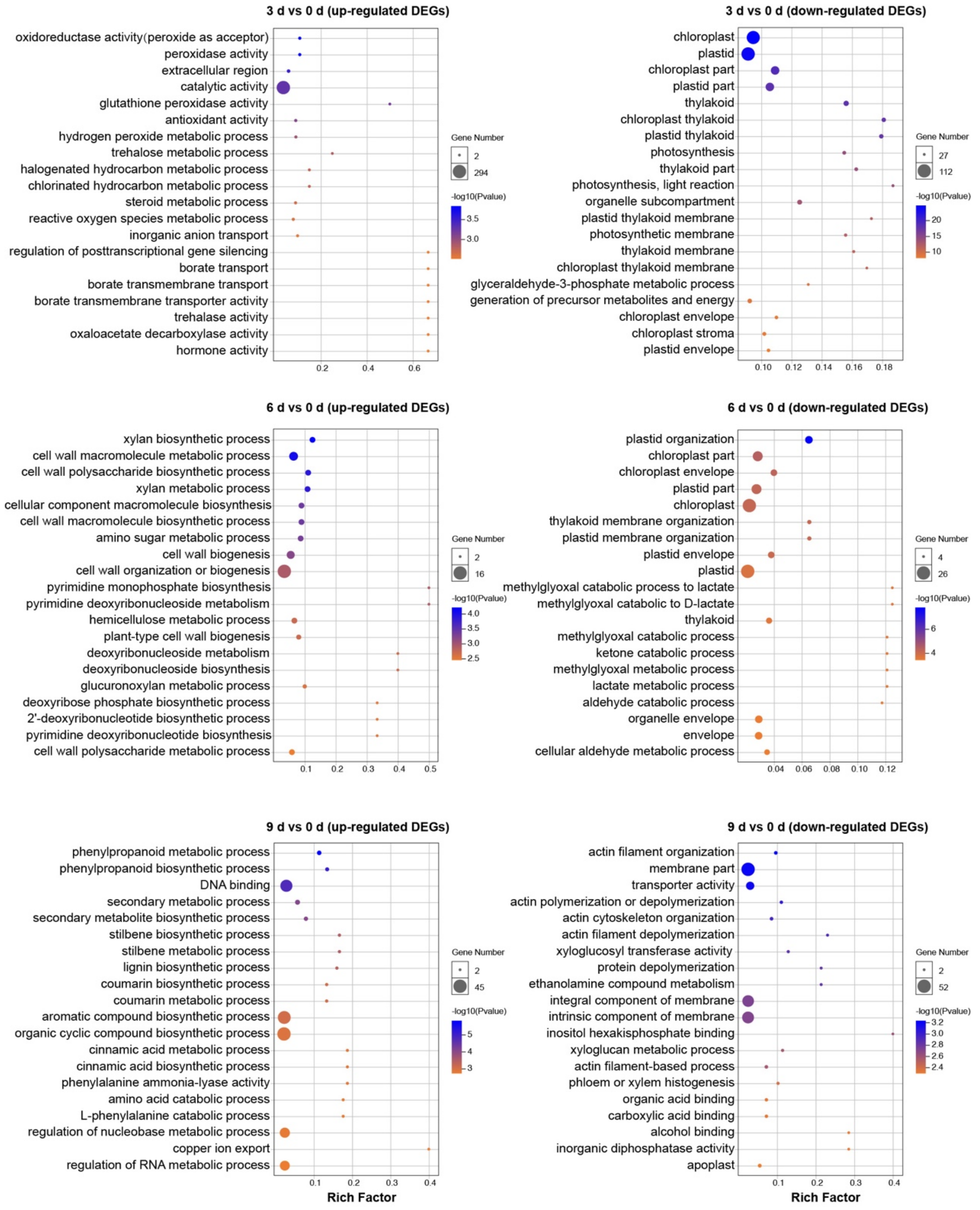

Fig. S6 Enriched GO categories for DEGs which were only significantly expressed in comparisons of 3 d vs 0 d, 6 d vs 0 d and 9 d vs 0 d in cucumber.

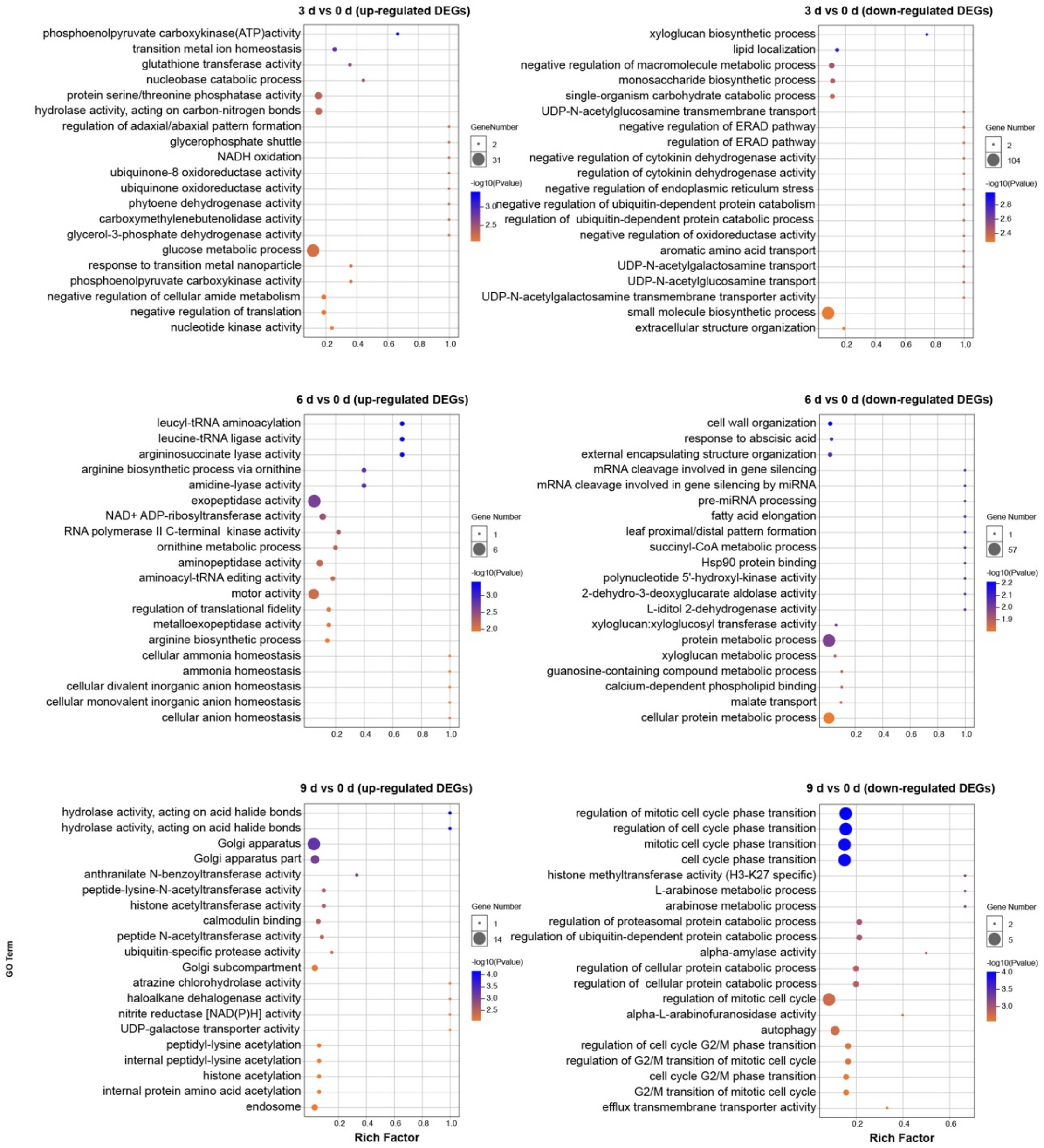

Fig. S7 Enriched GO categories for DEGs which were only significantly expressed in comparisons of 3 d vs 0 d, 6 d vs 0 d and 9 d vs 0 d in pumpkin.

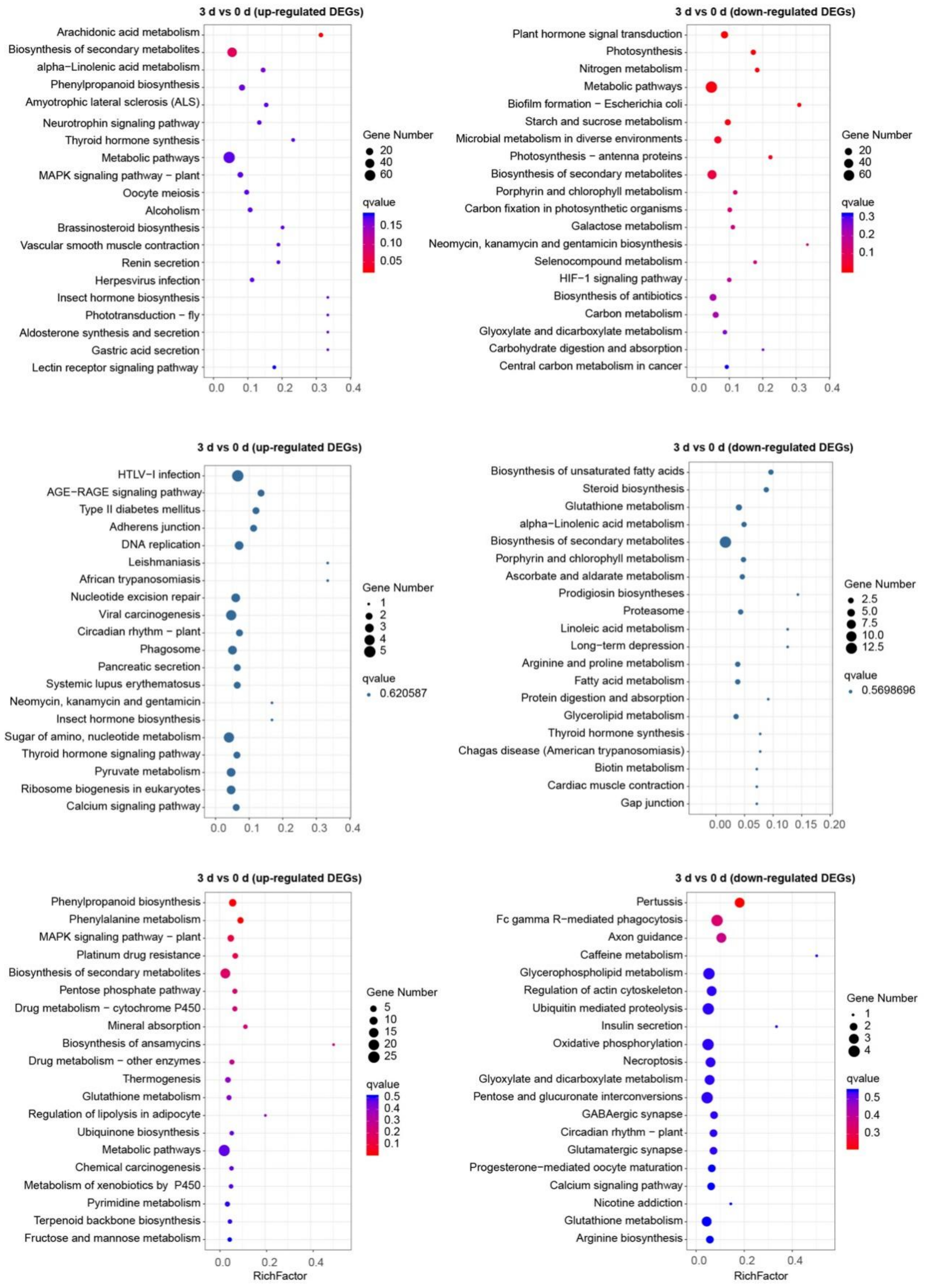

Fig. S8 Enriched KEGG pathways of DEGs which were only significantly expressed in comparisons of 3 d vs 0 d, 6 d vs 0 d and 9 d vs 0 d in cucumber.

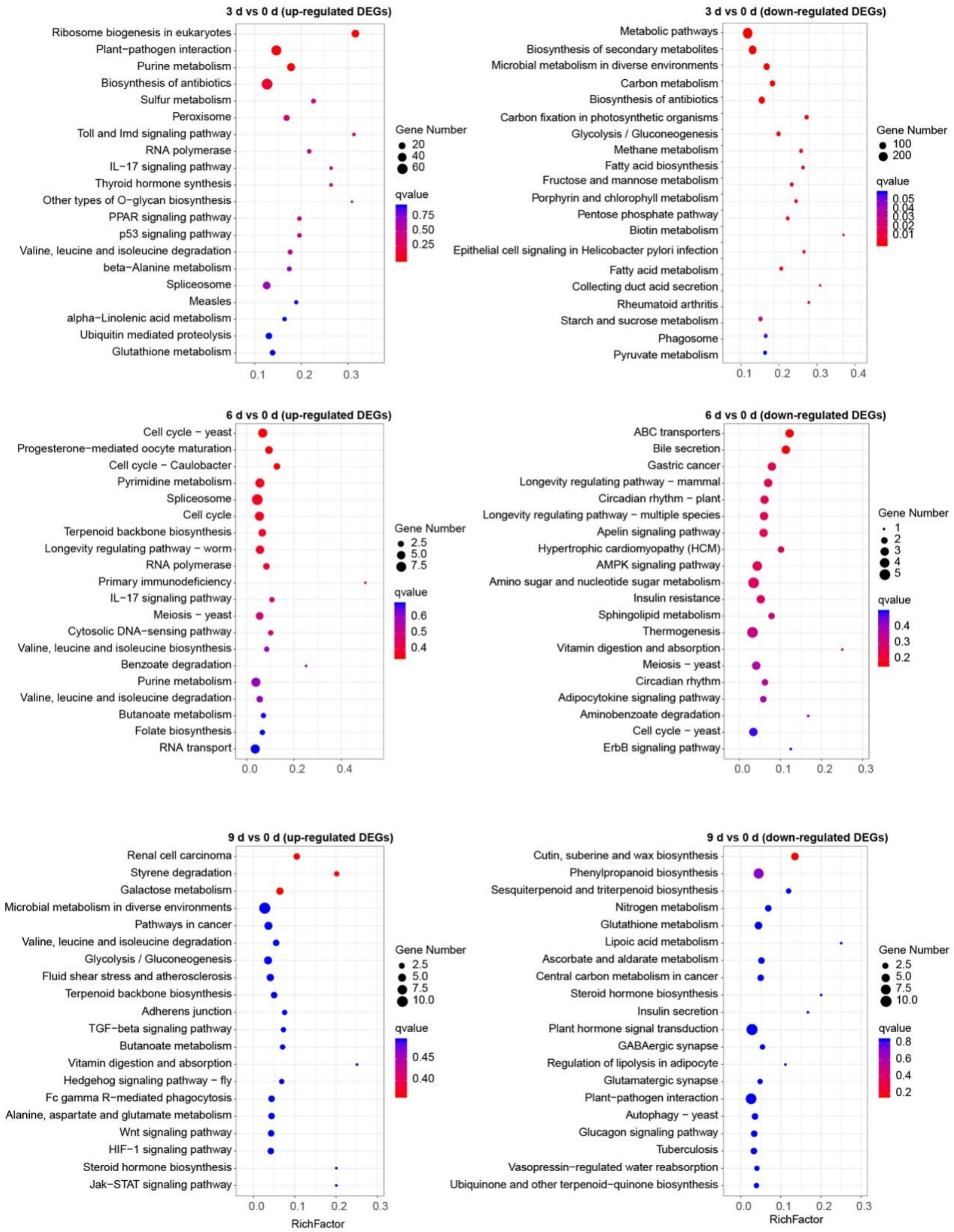

Fig. S9 Enriched KEGG pathways of DEGs which were only significantly expressed in comparisons of 3 d vs 0 d, 6 d vs 0 d and 9 d vs 0 d in pumpkin.

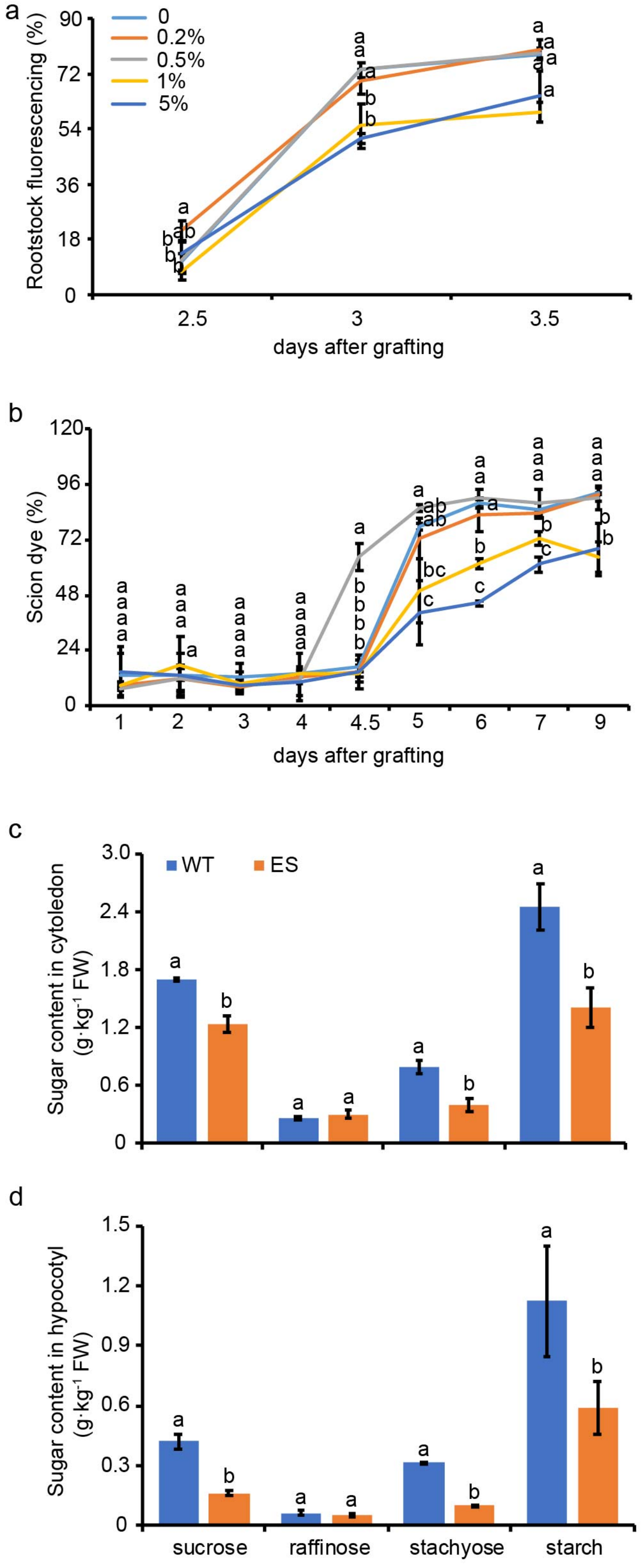

Fig. S10 Effect of glucose on the graft union formation and the levels of sugars in normal cucumber and etiolated cucumber. a, b phloem and xylem reconnection was monitored daily as described in **Fig. S1b** after spraying 0, 0.2, 0.5, 1, 5 % glucose. **c, d** The levels of sucrose, raffinose, stachyose, and starch in the cotyledon and hypocotyl of etiolated seedling (ES) and normal cucumber (WT), respectively. Error bars indicate SE (n=3). Different letters indicate significant differences (one-way ANOVA, $P < 0.05$).

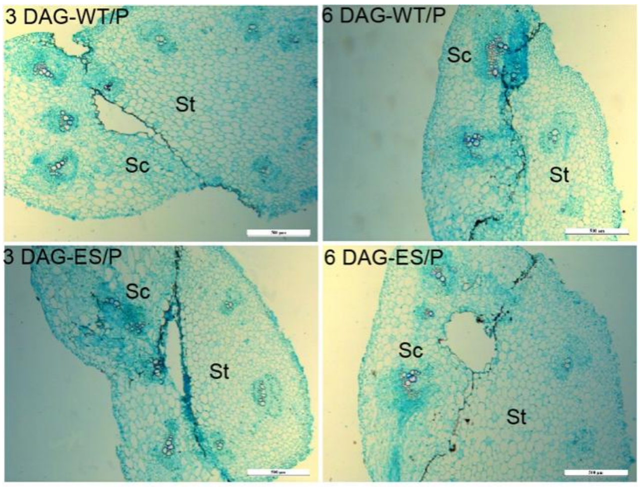

Fig. S11 Transverse sections of graft union at 3 DAG and 6 DAG in WT/P and ES/P, respectively. DAG, days after grafting; WT/P, the normal cucumber grafted onto pumpkin; ES/P, the etiolated cucumber grafted onto pumpkin. Data represent the means of 5 replicates ± SE.

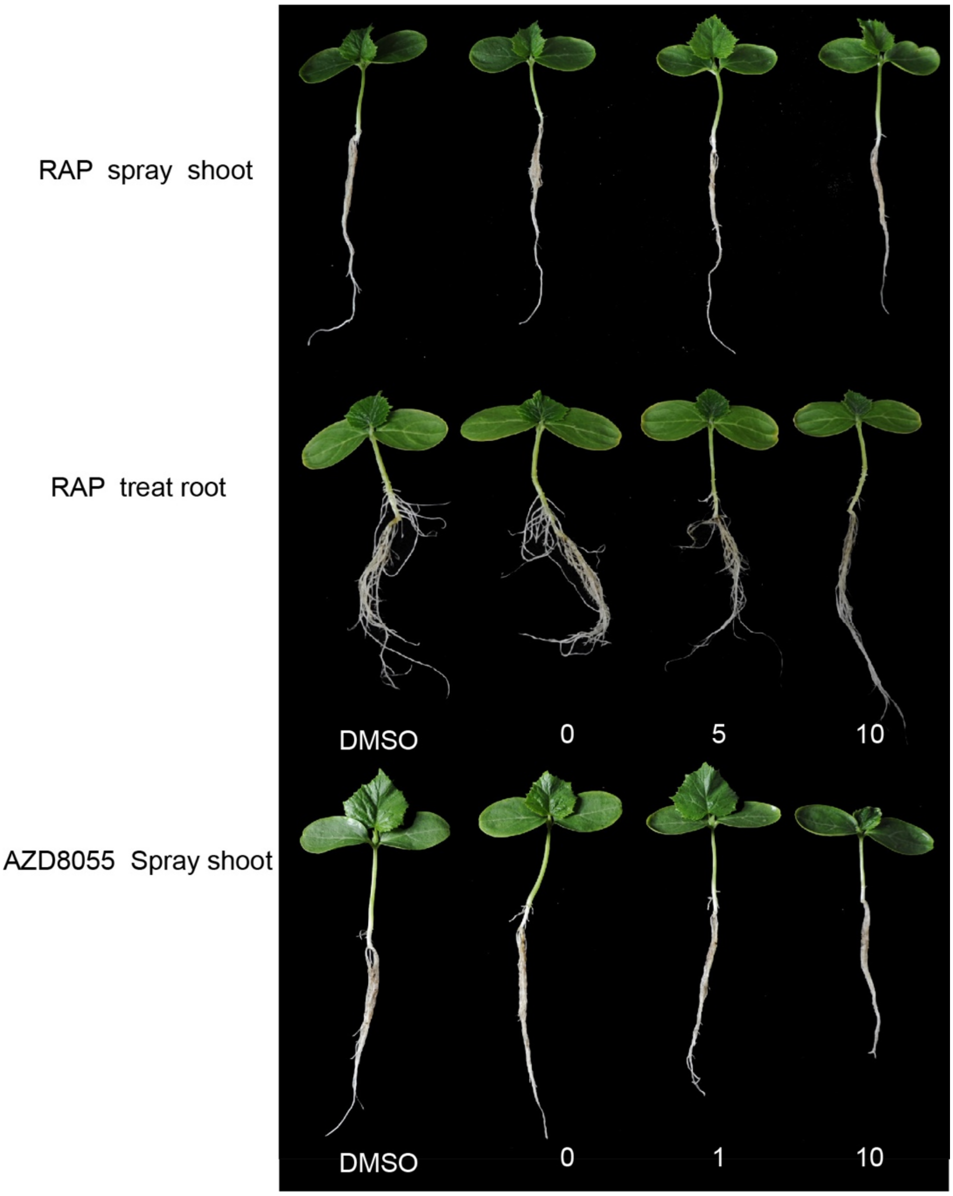

Fig. S12 Effect of rapamycin and AZD-8055 on the cucumber growth. The cucumber was sprayed by various concentration of rapamycin (0, 5, 10 μM) or added it in the nutrient solution. The cucumber was sprayed by various concentration of AZD8055 (0, 1, 10 μM).

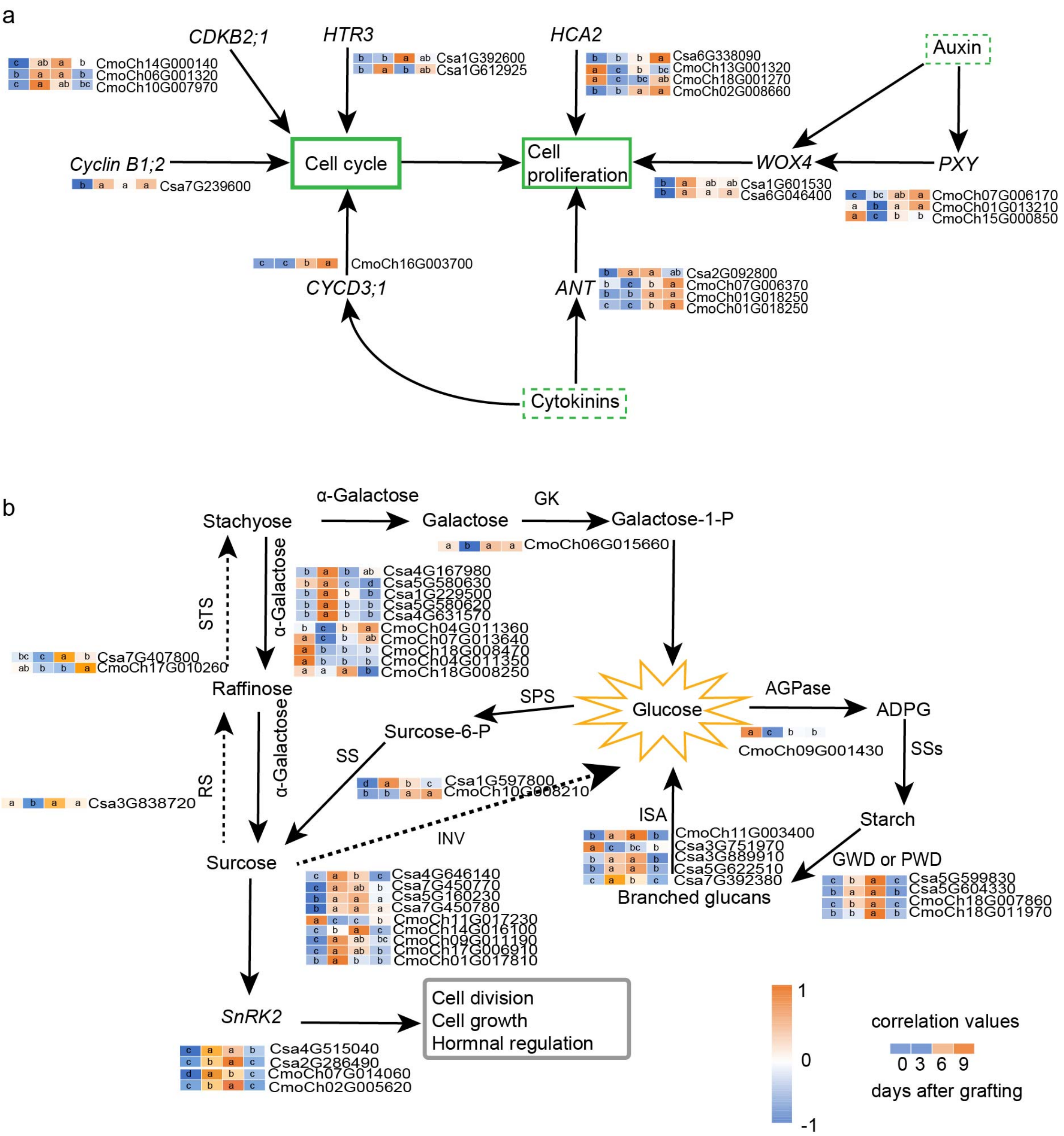

Fig. S13 Overview of expression patterns of DEGs in genes related to cambium development and cell division (a) and sugar metabolism (b) at the graft junction. S-AI, soluble acid invertase; NI, neutral invertase; SS, sucrose synthase; SPS, Sucrose phosphate synthase; GAL, α-galactosidase; SSS, Soluble starch synthase; SBE, Starch branching enzyme; DEGs, different expression genes; DAG, days after grafting; GK, galactokinase; SPS, sucrose phosphate synthase; SS, sucrose synthase; SSs, starch synthase; GWD, glucan water dikinase; PWD, phosphoglucan water dikinase; ISA, isoamylase; INV, intertase. Different letters indicate significant differences (one-way ANOVA, $P < 0.05$).

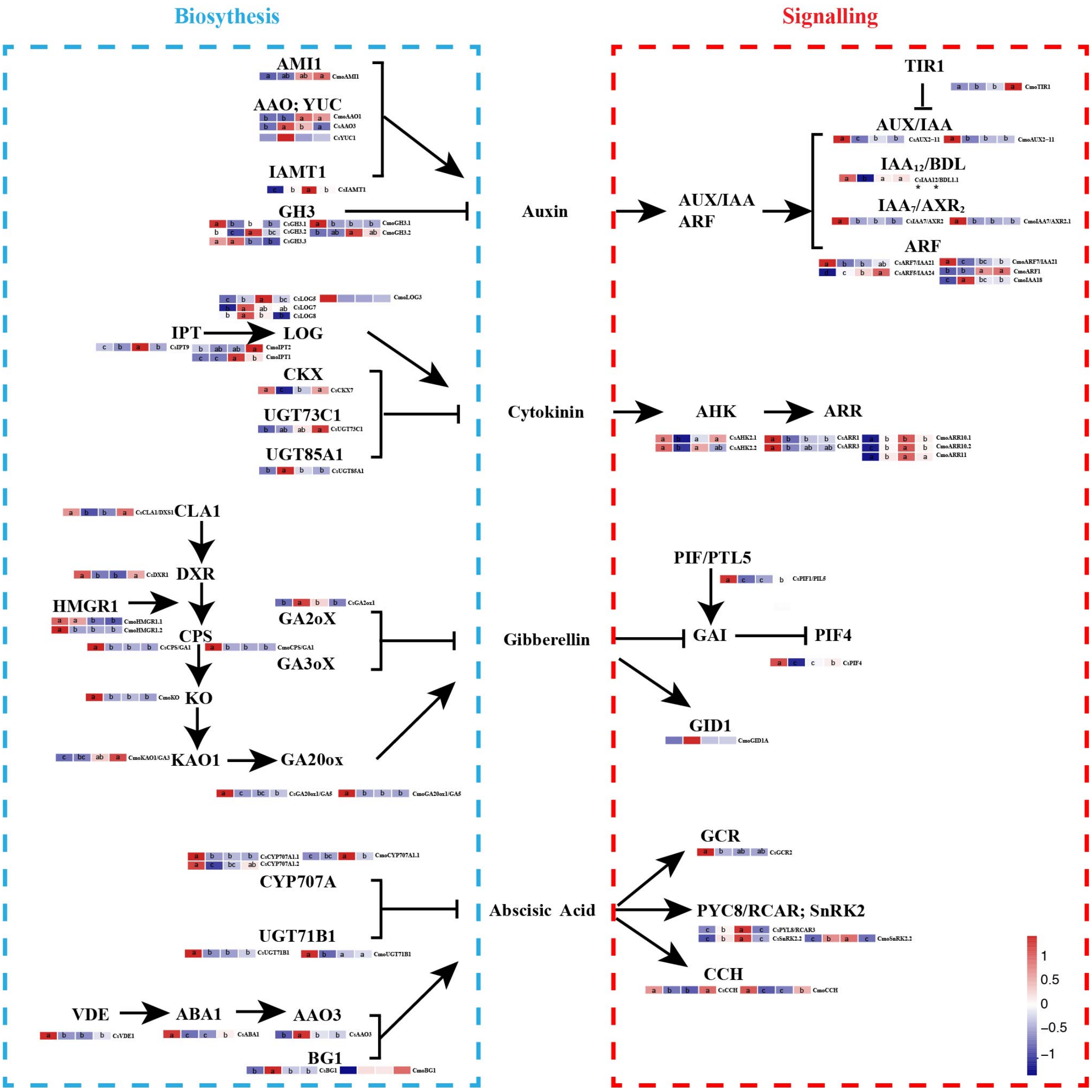

Fig. S14 Expression patterns of hormonal signaling-related DEGs at graft union formation. Different letters indicate significant differences (one-way ANOVA, $P < 0.05$).

Table S2. The statistical analysis of data in Fig. 4e.

Gene name	Treatments	0 DAG	0.5 DAG	1 DAG	1.5 DAG	2 DAG	3 DAG	4 DAG	5 DAG	6 DAG	7 DAG	9 DAG
CsTOR	WT/P	1.000^{bc}	0.391^{f}	0.345^{f}	0.530^{f}	0.385^{f}	0.742^{de}	0.880^{bcd}	1.074^{b}	0.826^{cd}	1.066^{b}	1.778^{a}
	WT/P+S	1.000^{cd}	0.625^{e}	0.659^{e}	0.840^{cde}	0.761^{de}	1.074^{c}	1.818^{b}	1.837^{b}	1.641^{b}	2.363^{a}	2.121^{a}
	ES/P	1.000^{a}	0.184^{gh}	0.212^{fgh}	0.145^{h}	0.187^{fg}	0.267^{ef}	0.440^{c}	0.338^{de}	0.233^{fg}	0.379^{cd}	0.759^{b}
	ES/P+S	1.000^{a}	0.321^{d}	0.287^{d}	0.282^{d}	0.402^{d}	0.523^{bc}	0.347^{d}	0.493^{bc}	0.531^{bc}	0.484^{c}	0.612^{b}
CmTOR	WT/P	1.000^{c}	0.465^{fg}	0.571^{ef}	0.356^{g}	0.574^{ef}	0.686^{de}	0.781^{d}	1.253^{b}	1.337^{ab}	1.441^{a}	1.486^{a}
	WT/P+S	1.000^{c}	0.513^{e}	0.602^{de}	0.690^{d}	1.001^{de}	0.942^{c}	1.547^{a}	1.060^{c}	1.031^{c}	1.554^{a}	1.216^{b}
	ES/P	1.000^{a}	0.386^{d}	0.376^{d}	0.211^{e}	0.202^{d}	0.532^{c}	0.670^{b}	0.587^{bc}	0.582^{bc}	0.585^{bc}	0.507^{cd}
	ES/P+S	1.000^{bc}	0.794^{cd}	0.590^{ef}	0.488^{f}	0.629^{ef}	1.288^{a}	0.855^{cd}	0.900^{cd}	0.807^{cd}	0.729^{de}	1.164^{ab}
CsS6K2	WT/P	1.000^{b}	0.402^{f}	0.333^{f}	0.454^{f}	0.384^{f}	0.651^{e}	0.712^{de}	0.920^{bc}	0.678^{de}	0.822^{cd}	1.320^{a}
	WT/P+S	1.000^{d}	0.830^{d}	1.029^{d}	0.906^{d}	1.001^{d}	1.478^{cd}	1.821^{c}	2.128^{c}	2.152^{c}	3.792^{a}	2.912^{b}
	ES/P	1.000^{a}	0.244^{f}	0.204^{f}	0.180^{f}	0.387^{b}	0.256^{f}	0.608^{bc}	0.376^{def}	0.538^{bcd}	0.461^{cde}	0.343^{ef}
	ES/P+S	1.000^{a}	0.297^{ef}	0.489^{bcde}	0.160^{f}	0.359^{de}	1.172^{a}	0.437^{bcde}	0.643^{bc}	0.612^{bcd}	0.678^{b}	0.430^{cde}
CmS6K2	WT/P	1.000^{c}	1.228^{b}	1.835^{a}	1.101^{bc}	1.725^{a}	0.843^{de}	0.626^{f}	0.788^{e}	0.726^{ef}	0.982^{cd}	0.808^{e}
	WT/P+S	1.000^{b}	0.719^{c}	0.594^{de}	0.686^{cd}	1.531^{a}	0.467^{fg}	0.541^{ef}	0.250^{i}	0.332^{hi}	0.917^{b}	0.414^{gh}
	ES/P	1.000^{ab}	1.257^{a}	1.119^{a}	0.958^{ab}	0.760^{bc}	0.511^{cd}	1.012^{ab}	1.119^{a}	0.484^{cd}	0.351^{d}	0.631^{cd}
	ES/P+S	1.000^{b}	1.048^{b}	1.009^{b}	0.842^{b}	0.358^{cd}	2.979^{a}	0.828^{b}	0.213^{d}	0.286^{d}	0.308^{d}	0.583^{c}
CsE2Fa	WT/P	1.000^{a}	0.471^{ef}	0.419^{f}	0.606^{d}	0.465^{ef}	0.719^{c}	0.841^{b}	0.824^{b}	0.707^{c}	0.511^{e}	0.540^{de}
	WT/P+S	1.000^{de}	0.897^{e}	1.502^{bc}	1.036^{de}	1.260^{cd}	1.212^{d}	1.984^{a}	1.971^{a}	2.132^{a}	1.675^{b}	1.190^{d}
	ES/P	1.000^{a}	0.326^{cd}	0.202^{d}	0.323^{cd}	0.429^{c}	0.376^{c}	0.860^{b}	0.428^{c}	0.385^{c}	0.314^{cd}	0.360^{c}

	ES/P+S	1.000^{a}	0.291^{c}	0.108^{d}	0.111^{d}	0.118^{d}	0.902^{a}	0.489^{b}	0.332^{c}	0.246^{cd}	0.203^{cd}	0.244^{cd}
CmE2Fa	WT/P	1.000^{a}	0.472^{d}	0.607^{bc}	0.718^{b}	0.529^{cd}	0.555^{bc}	0.644^{bc}	0.611^{c}	0.595^{cd}	0.528^{cd}	0.541^{cd}
	WT/P+S	1.000^{a}	0.463^{e}	0.493^{de}	0.582^{c}	0.716^{b}	0.420^{f}	0.327^{cd}	0.554^{f}	0.308^{f}	0.345^{f}	0.297^{g}
	ES/P	1.000^{a}	0.459^{b}	0.380^{bc}	0.182^{d}	0.467^{b}	0.243^{cd}	0.288^{d}	0.410^{b}	0.239^{d}	0.453^{e}	0.062^{d}
	ES/P+S	1.000^{b}	0.727^{c}	0.410^{d}	0.244^{d}	0.695^{c}	0.442^{c}	1.868^{a}	0.667^{c}	1.037^{b}	0.352^{d}	0.393^{d}
*CsSnRk1*α	WT/P	1.000^{ab}	0.565^{f}	0.568^{f}	0.691^{ef}	0.706^{def}	0.753^{de}	0.700^{def}	0.912^{bc}	0.832^{cd}	1.046^{a}	1.100^{a}
	WT/P+S	1.000^{b}	0.715^{cde}	0.569^{e}	0.654^{de}	0.860^{bcd}	0.913^{bc}	1.042^{ab}	0.955^{b}	1.047^{ab}	1.252^{a}	1.084^{ab}
	ES/P	1.000^{f}	1.314^{de}	1.118^{ef}	1.151^{ef}	1.640^{c}	2.270^{b}	2.649^{a}	1.675^{c}	1.551^{cd}	1.732^{c}	1.321^{de}
	ES/P+S	1.000^{ef}	0.977^{ef}	0.844^{f}	1.389^{bcd}	1.095^{def}	1.155^{def}	2.116^{a}	1.543^{b}	1.217^{cde}	1.490^{bcd}	0.902^{ef}
*CmSnRk1*α	WT/P	1.000^{b}	0.477^{d}	0.725^{c}	0.602^{cd}	0.762^{c}	0.703^{c}	0.607^{cd}	0.710^{c}	0.700^{c}	1.295^{a}	0.723^{c}
	WT/P+S	1.000^{a}	0.384^{ef}	0.403^{de}	0.488^{cd}	0.717^{b}	0.501^{c}	0.698^{b}	0.371^{ef}	0.547^{c}	0.777^{b}	0.302^{f}
	ES/P	1.000^{e}	1.275^{de}	1.214^{e}	1.100^{e}	0.923^{e}	2.496^{a}	1.987^{b}	1.607^{cd}	1.152^{e}	1.836^{bc}	1.557^{cd}
	ES/P+S	1.000^{cd}	0.855^{cde}	0.967^{cde}	0.702^{e}	1.260^{b}	1.825^{a}	1.091^{bc}	0.865^{cde}	0.792^{de}	0.855^{cde}	0.905^{cde}
CsSnRK2.1	WT/P	1.000^{e}	1.294^{d}	0.719^{f}	1.214^{d}	0.702^{f}	5.230^{a}	1.320^{d}	2.227^{c}	2.862^{b}	1.301^{d}	2.159^{c}
	WT/P+S	1.000^{b}	0.900^{bcd}	0.319^{f}	0.285^{f}	0.893^{bcd}	0.531^{e}	0.793^{d}	0.634^{e}	0.982^{bc}	1.261^{a}	0.856^{cd}
	ES/P	1.000^{e}	3.059^{c}	2.170^{d}	3.501^{b}	2.111^{d}	1.806^{d}	1.082^{e}	4.409^{a}	3.646^{b}	3.358^{bc}	0.993^{e}
	ES/P+S	1.000^{b}	0.957^{b}	1.147^{b}	0.111^{c}	0.169^{c}	5.187^{a}	0.158^{c}	0.174^{c}	0.206^{c}	0.043^{c}	0.269^{c}
CmSnRK2.1	WT/P	1.000^{d}	3.971^{a}	2.141^{b}	1.075^{d}	0.458^{e}	1.770^{c}	0.008^{f}	0.297^{e}	1.103^{d}	1.233^{d}	1.035^{d}
	WT/P+S	1.000^{ef}	0.720^{fg}	0.577^{g}	1.758^{b}	1.436^{cd}	1.246^{de}	2.852^{a}	0.792^{fg}	1.716^{bc}	0.825^{fg}	1.428^{cd}
	ES/P	1.000^{g}	56.671^{a}	46.118^{b}	25.401^{c}	3.693^{f}	5.228^{f}	4.009^{f}	10.412^{d}	7.405^{e}	3.786^{f}	2.936^{fg}
	ES/P+S	1.000^{e}	1.875^{cd}	0.196^{f}	1.122^{d}	4.526^{a}	2.368^{b}	2.110^{bc}	0.125^{f}	1.575^{d}	0.096^{f}	0.460^{f}
CsSnRK2.2	WT/P	1.000^{c}	1.263^{b}	0.139^{g}	0.613^{d}	0.301^{f}	1.532^{a}	0.007^{h}	0.383^{f}	0.507^{e}	1.598^{a}	1.007^{c}

	WT/P+S	1.000^{d}	0.451^{f}	0.166^{gh}	0.131^{h}	0.699^{e}	0.343^{fg}	1.932^{a}	0.835^{de}	0.796^{e}	1.554^{b}	1.254^{c}
	ES/P	1.000^{g}	28.567^{a}	25.901^{b}	13.890^{c}	4.408^{f}	7.342^{e}	10.096^{d}	14.210^{c}	14.542^{c}	6.460^{ef}	1.078^{g}
	ES/P+S	1.000^{b}	0.337^{cd}	1.079^{b}	1.066^{b}	0.389^{c}	2.767^{a}	0.094^{e}	0.381^{c}	0.155^{e}	0.222^{cde}	0.173^{de}
CmSnRK2.2	WT/P	1.000^{f}	1.928^{a}	1.780^{b}	1.870^{ab}	1.682^{c}	1.552^{d}	0.012^{i}	0.218^{h}	0.655^{g}	1.347^{e}	0.580^{f}
	WT/P+S	1.000^{ef}	1.978^{b}	1.309^{cd}	1.934^{b}	2.267^{a}	1.429^{c}	1.571^{c}	1.121^{de}	0.800^{f}	0.750^{f}	0.996^{ef}
	ES/P	1.000^{fg}	17.614^{a}	16.384^{b}	7.406^{c}	1.402^{ef}	1.929^{e}	3.408^{d}	3.019^{d}	1.395^{ef}	1.379^{ef}	0.317^{g}
	ES/P+S	1.000^{c}	0.916^{c}	1.216^{b}	1.689^{a}	1.740^{a}	0.868^{c}	0.237^{ef}	0.315^{e}	0.123^{f}	0.267^{ef}	0.522^{d}

Note: Different letters indicate significant differences among different time points (one-way ANOVA, $P < 0.05$). WT/P, the normal cucumber grafted onto pumpkin; WT/P+S, the normal cucumber grafted onto pumpkin under 0.5% glucose treatment; ES/P, the etiolated cucumber grafted onto pumpkin; ES/P+S, the etiolated cucumber grafted onto pumpkin under 0.5% glucose treatment. DAG, days after grafting.

Table S3. The statistical analysis of data in Fig. 5d.

Items	Treatments	0 DAG	1 DAG	3 DAG	6 DAG	9 DAG
ATP	WT/PSc	9.58^{a}	14.73^{a}	6.79^{a}	5.76^{a}	4.62^{a}
($\mu g \cdot g^{-1}$ FW)	WT/P+AZDSc	9.54^{a}	11.51^{b}	2.73^{b}	3.24^{b}	3.92^{ab}
	WT/P+AZD+SSc	9.24^{a}	13.87^{a}	3.15^{b}	3.15^{b}	3.55^{b}
	WT/PSt	8.69^{a}	5.37^{a}	7.33^{a}	6.21^{a}	5.00^{ab}
	WT/P+AZDSt	7.97^{a}	4.19^{c}	7.00^{a}	4.54^{b}	5.52^{a}
	WT/P+AZD+SSt	7.64^{a}	4.66^{b}	6.55^{a}	5.63^{a}	4.44^{b}
ADP	WT/PSc	9.45^{a}	10.68^{a}	1.34^{b}	1.25^{c}	1.99^{ab}
($\mu g \cdot g^{-1}$ FW)	WT/P+AZDSc	9.56^{a}	10.60^{a}	1.83^{b}	2.26^{b}	2.22^{a}
	WT/P+AZD+SSc	9.89^{a}	10.27^{a}	3.02^{a}	3.02^{a}	1.81^{b}
	WT/PSt	7.09^{a}	2.56^{a}	3.98^{a}	1.68^{b}	2.33^{b}

	WT/P+AZDSt	7.80^{a}	2.40^{a}	3.25^{c}	2.34^{a}	3.09^{a}
	WT/P+AZD+SSt	7.80^{a}	1.61^{b}	3.43^{b}	1.94^{ab}	1.62^{c}
AMP	WT/PSc	7.85^{a}	2.87^{a}	1.55^{c}	4.50^{b}	3.90^{a}
(μg·g^{-1} FW)	WT/P+AZDSc	7.10^{a}	3.20^{a}	4.18^{a}	7.86^{a}	3.35^{a}
	WT/P+AZD+SSc	7.34^{a}	3.92^{a}	2.97^{b}	2.97^{c}	3.14^{a}
	WT/PSt	6.13^{a}	1.97^{b}	4.45^{b}	5.41^{b}	2.66^{b}
	WT/P+AZDSt	6.82^{a}	2.39^{a}	6.23^{a}	3.22^{c}	3.24^{a}
	WT/P+AZD+SSt	6.16^{a}	2.39^{a}	4.47^{b}	6.11^{a}	2.90^{ab}
Ec value	WT/PSc	0.53^{a}	0.71^{a}	0.77^{a}	0.56^{a}	0.53^{a}
	WT/P+AZDSc	0.55^{a}	0.66^{a}	0.42^{c}	0.33^{c}	0.53^{a}
	WT/P+AZD+SSc	0.54^{a}	0.68^{a}	0.51^{b}	0.51^{b}	0.52^{a}
	WT/PSt	0.56^{a}	0.67^{a}	0.59^{a}	0.53^{ab}	0.62^{a}
	WT/P+AZDSt	0.52^{a}	0.60^{c}	0.52^{b}	0.56^{a}	0.60^{a}
	WT/P+AZD+SSt	0.53^{a}	0.63^{b}	0.57^{a}	0.48^{b}	0.59^{a}

Note: Different letters indicate significant differences among different treatments at same time points (one-way ANOVA, $P < 0.05$). WT/PSc, the scion of the normal cucumber grafted onto pumpkin; WT/PSt, the rootstock of the normal cucumber grafted onto pumpkin; WT/P+AZDSc, the scion of the normal cucumber grafted onto pumpkin under AZD8055 treatment; WT/P+AZDSt, the rootstock of the normal cucumber grafted onto pumpkin under AZD8055 treatment; WT/P+AZD+SSc, the scion of the normal cucumber grafted onto pumpkin under AZD8055 and 0.5% glucose treatment; WT/P+AZD+SSt, the rootstock of the normal cucumber grafted onto pumpkin under AZD8055 and 0.5% glucose treatment. DAG, days after grafting.

Table S4. The statistical analysis of data in Fig. 6a.

Gene name	Treatments	0 DAG	0.5 DAG	1 DAG	1.5 DAG	2 DAG	3 DAG	4 DAG	5 DAG	6 DAG	7 DAG	9 DAG
CsTOR	WT/P	1.000^{bc}	0.391^{f}	0.345^{f}	0.530^{f}	0.385^{f}	0.742^{de}	0.880^{bcd}	1.074^{b}	0.826^{cd}	1.066^{b}	1.778^{a}

	WT/P+AZD	1.000^{cd}	0.820^{cde}	0.925^{cde}	0.710^{e}	0.830^{cde}	2.502^{a}	0.779^{cde}	1.390^{b}	1.040^{c}	1.360^{b}	0.731^{de}
	WT/P+AZD+S	1.000^{d}	0.460^{f}	0.452^{f}	0.627^{e}	0.709^{e}	1.070^{d}	0.770^{e}	1.559^{b}	1.357^{c}	1.752^{a}	1.639^{ab}
CmTOR	WT/P	1.000^{c}	0.465^{fg}	0.571^{ef}	0.356^{g}	0.574^{ef}	0.686^{de}	0.781^{de}	1.253^{b}	1.337^{ab}	1.441^{a}	1.486^{a}
	WT/P+AZD	1.000^{b}	0.333^{f}	0.511^{d}	0.484^{de}	0.306^{f}	0.811^{c}	0.351^{f}	0.382^{ef}	1.244^{a}	1.143^{a}	0.784^{c}
	WT/P+AZD+S	1.000^{a}	0.386^{f}	0.223^{g}	0.376^{f}	0.375^{f}	0.462^{cd}	0.393^{def}	0.596^{c}	0.471^{d}	0.804^{b}	0.468^{d}
CsS6K2	WT/P	1.000^{b}	0.402^{f}	0.333^{f}	0.454^{f}	0.384^{f}	0.651^{e}	0.712^{de}	0.920^{bc}	0.678^{de}	0.822^{cd}	1.320^{a}
	WT/P+AZD	1.000^{d}	0.354^{h}	0.589^{fg}	0.517^{gh}	0.786^{def}	1.832^{a}	0.840^{de}	1.503^{b}	1.674^{ab}	1.270^{c}	0.665^{efg}
	WT/P+AZD+S	1.000^{cd}	0.593^{fg}	0.528^{g}	0.710^{f}	0.837^{e}	1.116^{c}	0.915^{de}	1.522^{b}	1.466^{b}	1.595^{b}	2.352^{a}
CmS6K2	WT/P	1.000^{de}	1.367^{bc}	2.052^{a}	1.348^{bc}	2.159^{a}	1.201^{cde}	1.034^{de}	1.577^{bc}	0.521^{f}	0.916^{e}	1.226^{cd}
	WT/P+AZD	1.000^{a}	0.595^{bc}	0.823^{ab}	0.435^{cd}	0.282^{cd}	0.982^{a}	0.215^{cd}	0.188^{d}	0.200^{cd}	0.175^{d}	0.592^{bc}
	WT/P+AZD+S	1.000^{a}	0.998^{a}	0.421^{cde}	0.510^{c}	0.381^{e}	0.447^{cde}	0.492^{cd}	0.366^{e}	0.358^{e}	0.813^{b}	0.395^{de}
CsE2Fa	WT/P	1.000^{a}	0.471^{ef}	0.419^{f}	0.606^{d}	0.465^{ef}	0.719^{c}	0.841^{b}	0.824^{b}	0.707^{c}	0.511^{e}	0.540^{de}
	WT/P+AZD	1.000^{a}	0.021^{f}	0.143^{cde}	0.164^{cde}	0.179^{cde}	0.383^{b}	0.116^{de}	0.150^{cde}	0.211^{c}	0.197^{cd}	0.103^{e}

	WT/P+AZD+S	1.000^{b}	0.481^{d}	0.498^{d}	0.699^{c}	0.806^{c}	1.133^{a}	0.692^{c}	1.024^{ab}	0.774^{c}	0.792^{c}	0.993^{b}
CmE2Fa	WT/P	1.000^{a}	0.472^{d}	0.607^{bc}	0.718^{b}	0.529^{cd}	0.644^{bc}	0.611^{bc}	0.595^{c}	0.528^{cd}	0.541^{cd}	0.582^{cd}
	WT/P+AZD	1.000^{a}	0.039^{f}	0.368^{cd}	0.265^{e}	0.434^{c}	0.391^{cd}	0.034^{f}	0.094^{f}	0.590^{b}	0.433^{c}	0.307^{de}
	WT/P+AZD+S	1.000^{a}	0.244^{de}	0.207^{ef}	0.493^{b}	0.402^{c}	0.217^{ef}	0.188^{f}	0.197^{ef}	0.223^{def}	0.271^{d}	0.207^{ef}
*CsSnRk1*α	WT/P	1.000^{ab}	0.565^{f}	0.568^{f}	0.691^{ef}	0.706^{def}	0.753^{de}	0.700^{def}	0.912^{bc}	0.832^{cd}	1.046^{a}	1.100^{a}
	WT/P+AZD	1.000^{a}	0.027^{g}	0.153^{cd}	0.095^{ef}	0.116^{de}	0.229^{b}	0.064^{fg}	0.190^{bc}	0.080^{ef}	0.150^{cd}	0.093^{ef}
	WT/P+AZD+S	1.000^{a}	0.410^{gh}	0.322^{i}	0.360^{hi}	0.453^{fg}	0.509^{ef}	0.505^{ef}	0.560^{de}	0.636^{d}	0.855^{b}	0.755^{c}
*CmSnRk1*α	WT/P	1.000^{b}	0.477^{d}	0.725^{c}	0.602^{cd}	0.762^{c}	0.703^{c}	0.607^{cd}	0.710^{c}	0.700^{c}	1.295^{a}	0.723^{c}
	WT/P+AZD	1.000^{b}	0.630^{d}	0.801^{c}	0.394^{ef}	0.508^{de}	1.404^{a}	0.382^{ef}	0.310^{f}	0.446^{ef}	0.970^{b}	0.978^{b}
	WT/P+AZD+S	1.000^{a}	0.599^{b}	0.222^{f}	0.251^{ef}	0.480^{cd}	0.321^{e}	0.430^{d}	0.211^{f}	0.324^{e}	0.527^{bc}	0.456^{cd}

Note: Different letters indicate significant differences among different time points (one-way ANOVA, $P < 0.05$). WT/P, the normal cucumber grafted onto pumpkin; WT/P+AZD, the normal cucumber grafted onto pumpkin under AZD-8055 treatment; WT/P+AZD+S, the normal cucumber grafted onto pumpkin under AZD-8055 and 0.5% glucose treatment. DAG, days after grafting.

Table S5. The genes number in Fig. S14.

Gene name	Gene name	Gene name	Gene name	Gene name	Gene name	Gene name	Gene name

CsYUC1	Csa2G375750	*CsARF7/IAA24*	Csa3G866510	*CsIPT3*	Csa7G392940	*CsLOG5*	Csa6G127300
CsCYP79B2	Csa5G223620	*CsCPS/GA1*	Csa6G410650	*CsIPT9*	Csa4G083690	*CsLOG7*	Csa3G778430
CsIAMT1	Csa7G081680	*CsGA20ox1/GA5.1*	Csa5G172270	*CsCKX7*	Csa4G647490	*CsLOG8*	Csa4G646190
CsGH3.1	Csa3G198490	*CsPIF1/PIL5*	Csa7G333400	*CsUGT73C1*	Csa3G743980	*CsABA1*	Csa2G277050
CsGH3.2	Csa3G431430	*CsPIF4.1*	Csa4G615240	*CsUGT85A1*	Csa3G889760	*CsAAO3*	Csa4G269120
CsGH3.3	Csa4G007100	*CsCYP707A1.1*	Csa4G639800	*CsAHK2.1*	Csa5G549170	*CsDXR1.1*	Csa1G004930
CsAUX2−11	Csa2G200440	*CsCYP707A1.2*	Csa4G056600	*CsAHK2.2*	Csa6G095330	*CsGA2ox1*	Csa4G075200
CsIAA12/BDL1.1	Csa2G170820	*CsCCH*	Csa4G165920	*CsARR1*	Csa3G165610	*CsGA3ox1/GA4*	Csa7G434970
CsIAA7/AXR2	Csa2G200420	*CsVDE1*	Csa2G083740	*CsARR3*	Csa5G603910	*CsUGT71B1*	Csa1G660200
CsARF7/IAA21	Csa2G000030	*CsBG1*	Csa1G660200	*CsCLA1/DXS1*	Csa6G067390	*CsGCR2*	Csa4G638330
CsPYL8/RCAR3	Csa5G139540	*CmoAMI1*	CmoCh01G011590	*CmoIAA7/AXR2.2*	CmoCh06G009490	*CmoKAO1/GA3*	CmoCh14G020100
CsSNRK2.2	Csa2G286490	*CmoAAO1.1*	CmoCh02G012060	*CmoARR10.1*	CmoCh01G017020	*CmoCYP707A1.1*	CmoCh04G006430
CmoLOG3	CmoCh06G003750	*CmoGH3.1*	CmoCh14G014260	*CmoARR10.2*	CmoCh09G004260	*CmoCYP707A1.2*	CmoCh11G013610
CmoIPT1	CmoCh18G010240	*CmoGH3.2*	CmoCh16G003520	*CmoARR11*	CmoCh12G000370	*CmoARF7/IAA21*	CmoCh05G014250
CmoIPT2	CmoCh12G007970	*CmoTIR1*	CmoCh07G002690	*CmoGA20ox1/GA5.1*	CmoCh02G010360	*CmoUGT71B1*	CmoCh10G008480
CmoIPT3	CmoCh08G003550	*CmoAUX2−11*	CmoCh12G011430	*CmoCCH*	CmoCh03G000620	*CmoARF1*	CmoCh04G007210
CmoUGT73C1	CmoCh14G002330	*CmoIAA7/AXR2.1*	CmoCh17G001860	*CmoKO*	CmoCh09G001950	*CmoIAA18*	CmoCh01G000950
CmoHMGR1.1	CmoCh05G009770	*CmoHMGR1.2*	CmoCh19G008360	*CmoCPS/GA1*	CmoCh19G000340	*CmoSNRK2.2*	CmoCh02G005620
CmoBG1	CmoCh10G008480						

Table S6. Genes primer used for RT-qPCR in graft union healing analysis.

Gene name	Genes abbreviation	Forward primer （5’- 3’）	Reverse primer （5’- 3’）
Csa7G239600	*CsaCyclin B1;2*	GGAATAACCGAGCGAGTGA	AGACGCTGAAGGTTCCACA
Csa7G030510	*CsaAPL3*	CGTCGTCTTCATCGTCC	TCGTGATCCCAAACCAG
Csa7G030510	*CsaAPL3.1*	CTCACGGAACCCAAACGA	CAGAATGATCGCCACCACA
Csa6G289740	*CsaHCA2*	CTAAGCTCCAACATCAATCA	GCCAAGGAAATCCCAAC
Csa6G153460	*CsaAPL3.2*	GTTATACTTGGAGGAGGAGCG	AGCGAGATGCCTATTGAGC
Csa6G046400	*CsaPXY*	TCCACCTGAGTTAGGCTCG	CTGAAGACAGGCAGGCAAG
Csa6G014590	*CsaRUL*	TTCCAGTCTTGCTGCTGT	ATTCATACCCTTCACCTCC
Csa4G515040	*CsaSnRK2.1*	GATGGCAGATGTATGGTCGTG	GCTCCCTTGGCAAGTTCTTT
Csa3G812740	*CsaWOX4*	GCCGCTACTCACCAAGGTA	ACAATCTCCGCCAACCC
Csa2G286490	*CsaSnRK2.2*	GGTTGCCAGGTTGATGAGA	TTGAAAGAAGAAGCGAGCC
Csa2G092800	*CsaANT*	ACCATCCTTACGCTTTCC	TCCAATGTCTCCTAATCCC
Csa1G601530	*CsaPXY*	TTCCAACTCCGAAGGC	ATCCCAACTCTGAAGAACG
Csa1G071870	*CsaSTP1*	TGGCGTTTGAGTTTAGGT	ATGCGGTCGGTATTTCT
Csa1G071870	*CsaSTP*	GATGGCGTTTGAGTTTAGGTG	GAGATGCGGTCGGTATTTCT
Csa7G070760	*CsTOR*	CCTCGTTTGCGACATCTGA	AATGTGCTGGCGGACAATA
Csa1G007860	*CsS6K2*	ATGCTTCTAACTTCCCTCTTGG	TCCTTCGCTCGCTTTCC
Csa6G454430	*CsE2Fa*	CCTCTGCCTCACTCCCATAC	CACCCTTCTGCTTTGATTTAGC

Csa6G077450	*CsSnRK1α*	ATGCTCTTCTATGTGGCACTCT	GCATTGTATCAGGTGGAGGC
CmoCh18G003190	*CmoSTP*	GCCAATCAGTCTGTGCC	ACTCAAACGCCATCCC
CmoCh18G003190	*CmoSTP1.1*	CTCCGGTGGTTATCGTTTC	CCGAGAACTCTGGTCGTGTA
CmoCh18G001270	*CmoHCA2*	TCGGTACTGGACCAAAGGC	GATGATGGATCTGGGAAATAGG
CmoCh17G002500	*CmoAPL3.2*	GGACGGTGGTAGCGATTGT	GAGCGAGATGCCGATTGA
CmoCh17G002480	*CmoAPL*	ACTACATTCCAAATGCCAGAAG	AATCGCTACCACCGTCCTC
CmoCh17G002480	*CmoAPL3.1*	CTTAGCTCTAACGGAACAGGG	GTCTGATAGTAGTCCGCACCC
CmoCh16G003700	*CmoCYCD3;1*	TAGAACATACCCATCAGACCCA	ACCTGTCGAAATAAGCAACG
CmoCh14G000140	*CmoCDKB12.2*	CAGAAACAGGGACTTACCGC	TCTTCCACCCAACACGACT
CmoCh11G006450	*CmoSTP1.3*	CTGTGCCTTTGTATCTATCCG	GACTTTGGTCGTGCCTCTG
CmoCh09G008680	*CmoDIN6.2*	GTCACCCTCACAATACGTTCT	GCCTCTGTCCCTTAGTGTCC
CmoCh08G011380	*CmoDIN6.1*	AGTTTCGCTTTCGTCCTCTT	ACCTTGAATTTCGCACCC
CmoCh07G014060	*CmoSnRK2.1*	GGCATACATAGCACCAGAGG	TGATGGATTCGGGACAAAG
CmoCh06G001320	*CmoCDKB12.1*	CTGTAGACCCTGCTAAACGG	CTGGGTGAAGAACCAAAGG
CmoCh05G001250	*CmoANT*	CATTTAGCACGCAAGAGG	ATCCCGCCATTATTGTT
CmoCh02G008660	*CmoHCA2*	ACATGGTGGGCGTGAT	GTACCTTGGCTGGGAGA
CmoCh02G005620	*CmoSnRK2.2*	AGGCTCGCTTCTTCTTTCA	CATTACATAGAGGGTCACTCCACA
CmoCh01G013210	*CmoPXY*	GGGAAGTTGCCGATGT	TGGAAAGGGTGATGGAG
CmoCh01G013210	*CmoPXY*	ATATCTGTAGGTGCAGTGGTAGG	TGTTTGTGGCGGCTTTT
CmoCh01G013180	*CmoRUL*	CTGTGGTGGGAAGGAAG	CAGGATTTGGCATTGAGA
CmoCh19G005520	*CmTOR*	TCTAGGAATAATGGGTGCTCTG	GATGATAACTCGCAAGGGAAG
CmoCh11G001310	*CmS6K2*	CCGGAGCCATGTATTCTGT	ATTCGCCTTCTCAAGGGTT

CmoCh03G011170	*CmE2Fa*	TAGTTCTTAGGAGTACGATGGGTC	CCTGATGGCTTCTGTTGCT
CmoCh08G009290	*CmSnRK1α*	CGCTTCTATGCGGCACTCT	CATTGTATCAGGTGGAGGCAC
